# Modeling current geographic distribution and future range shifts of *Sanghuangporus* under multiple climate change scenarios in China

**DOI:** 10.3389/fmicb.2022.1064451

**Published:** 2022-12-01

**Authors:** Jia-He Chen, Shan Shen, Li-Wei Zhou

**Affiliations:** ^1^State Key Laboratory of Mycology, Institute of Microbiology, Chinese Academy of Sciences, Beijing, China; ^2^College of Life Science, Liaoning University, Shenyang, China; ^3^University of Chinese Academy of Sciences, Beijing, China

**Keywords:** conservation, fruitbodies, maximum entropy modeling, medicinal resources, wood-inhabiting macrofungi

## Abstract

The genus *Sanghuangporus* is well-known for its edible and medicinal values. In this study, the most comprehensive occurrence records of *Sanghuangporus* with accurate species identification are subjected to MaxEnt, to model the current geographic distribution and future range shifts under multiple climate change scenarios in China. The current potential distribution model of *Sanghuangporus* is excellently predicted as indicated by the value of Area Under Receiver Operator Characteristic Curve. The current potential distribution basically corresponds to the known occurrence records of *Sanghuangporus*, and provides clues to new suitable habitats. The critical environmental variables to the distribution are annual precipitation, host plant, annual mean temperature and elevation. Host plant is not the most critical contribution to the model, but it indeed plays a decisive role in restricting the distribution of *Sanghuangporus*. This role is further confirmed by the distribution area of the highly suitable habitat increasing by 155.468%, when excluding host plant from environmental variables. For future scenarios, generally the area of highly suitable habitat for *Sanghuangporus* extremely increases, but the locations do not change a lot. In conclusion, this study provides important ecological information for the utilization and conservation of the edible and medicinal fungus *Sanghuangporus*.

## Introduction

Macrofungi are a group of fungal species that produce fruitbodies visible to the naked eye. These fungi, commonly called ‘mushrooms’, have been used as edible and medicinal food around world, and in China for thousands of years (Yuan et al., 2018). Macrofungi have been found to have nutritional and medicinal benefits for humans (Wu et al., 2019a). The fleshy fruitbodies are prepared like other foods, while the tough ones are ground into powders and used as supplements to food or tea-like drinks (Wu et al., 2019a; Cheng et al., 2022; Zhou et al., 2022). In China, the consumption of wild and cultivated fruitbodies of macrofungi is a large business expanding quickly. Of macrofungi, species in *Sanghuangporus* have attracted more and more attention from scientific research and industry development (Zhou et al., 2022). These species, recorded as ‘Sanghuang’ in the ancient books of traditional Chinese medicines, were recently identified to the fungal genus *Sanghuangporus* in *Hymenochaetales*, *Basidiomycota* (Wu et al., 2012; Zhou et al., 2016), which further facilitates the medicinal utilization of these fungal resources (Zhou, 2020). For now, 18 species are named in *Sanghuangporus* (Wu et al., 2022), and 10 of them, *viz.*, *S. alpinus*, *S. baumii*, *S. lonicericola*, *S. quercicola*, *S. sanghuang*, *S. subbaumii*, *S. vaninii*, *S. vitexicola*, *S. weigelae*, and *S. zonatus* are distributed in China (Wu et al., 2020; Shen et al., 2021; Zhou et al., 2022). Since the publication of the first modern scientific research of ‘Sanghuang’ having antitumor properties (Ikekawa et al., 1968), this group of fungi has been intensively subjected to medicinal studies, especially in China. Besides antitumor properties, ‘Sanghuang’ has also been shown to possess other medicinal functions such as antioxidant, antidiabetic activity, anti-inflammation, immunomodulation, and hepatoprotection (He et al., 2021; Hou et al., 2021; Zhou et al., 2022).

Due to the above-mentioned health benefits, the demand for fruitbodies of *Sanghuangporus* in China increases year by year. Of the 10 species of *Sanghuangporus* in China, only *S. baumii* and *S. vaninii* are cultivated on a large scale. *Sanghuangporus sanghuang* has been cultivated in the laboratory, while the cultivation of other seven species has never been reported (Yang et al., 2022).

Different species of *Sanghuangporus* inhabit various host plants with weak or strong specificity and occupy different ecological niches (Wu et al., 2020; Shen et al., 2021), and perhaps, a certain species may possess specific medicinal functions. The valuable medicinal resource of these uncultivated species of *Sanghuangporus* should not be ignored. Furthermore, as consumers prefer to pick from the wild, the distribution knowledge of *Sanghuangporus* in the wild is important for effective utilization and conservation of these fungal resources in China.

Species distribution models (SDMs) are widely used for predicting potential geographic distribution of various life forms based on currently known distribution in association with various environmental variables of these locations (Elith and Leathwick, 2009; Zurell et al., 2020). For the past two decades, SDMs have been increasingly used for modeling fungi (Hao et al., 2020). One of the modeling methods, maximum entropy (MaxEnt) modeling is characterized by a data-friendly algorithm (Phillips et al., 2006). Maybe due to this character being suitable for fungi with poor knowledge of species diversity (Hawksworth and Lücking, 2017), MaxEnt modeling seems to be the most popular prediction method of species distribution for fungi (Banasiak et al., 2019; Guo et al., 2019; Bie et al., 2021; Freestone et al., 2021; Pietras et al., 2021; Wei et al., 2021; Yu et al., 2021) as well as for other life forms (Li et al., 2020; Qin et al., 2020; Tang et al., 2021; Zhan et al., 2022).

The potential distribution of ‘Sanghuang’ was predicted by Yuan et al. (2015); however, the taxonomic system of ‘Sanghuang’ was not well established at that time. Of the three predicted species in Yuan et al. (2015), *Phellinus igniarius* is excluded from ‘Sanghuang’ (Zhou et al., 2016), and some records of another two species *Phellinus baumii* (= *S. baumii*) and *P. vaninii* (= *S. vaninii*) may represent *S. alpinus*, *S. quercicola*, *S. subbaumii*, *S. weigelae* and other morphology-similar species (Shen et al., 2021). It is a common sense that the well-established systematics is crucial for utilization and conservation of fungal resources (Zhou, 2020; Zhou and May, 2022). Moreover, the information on host plants that directly and strictly restricts the distribution of ‘Sanghuang’ (Wu et al., 2020; Shen et al., 2021; Zhou et al., 2022) was not considered by Yuan et al. (2015). Given the above, the geographic distribution of ‘Sanghuang’, the important edible and medicinal wood-inhabiting fungi both culturally and economically (Zhou et al., 2022), deserves a more precise and updated modeling.

In this study, the most comprehensive and accurately identified species records of *Sanghuangporus* with related ecological information to date were subjected to MaxEnt modeling the current geographic distribution of the genus *Sanghuangporus* in its entirety in China. Moreover, the effects of host plants on the geographic distribution and the shifts of future ranges under multiple climate change scenarios were tested.

## Materials and methods

### Fungal occurrence records

**Figure 1:**
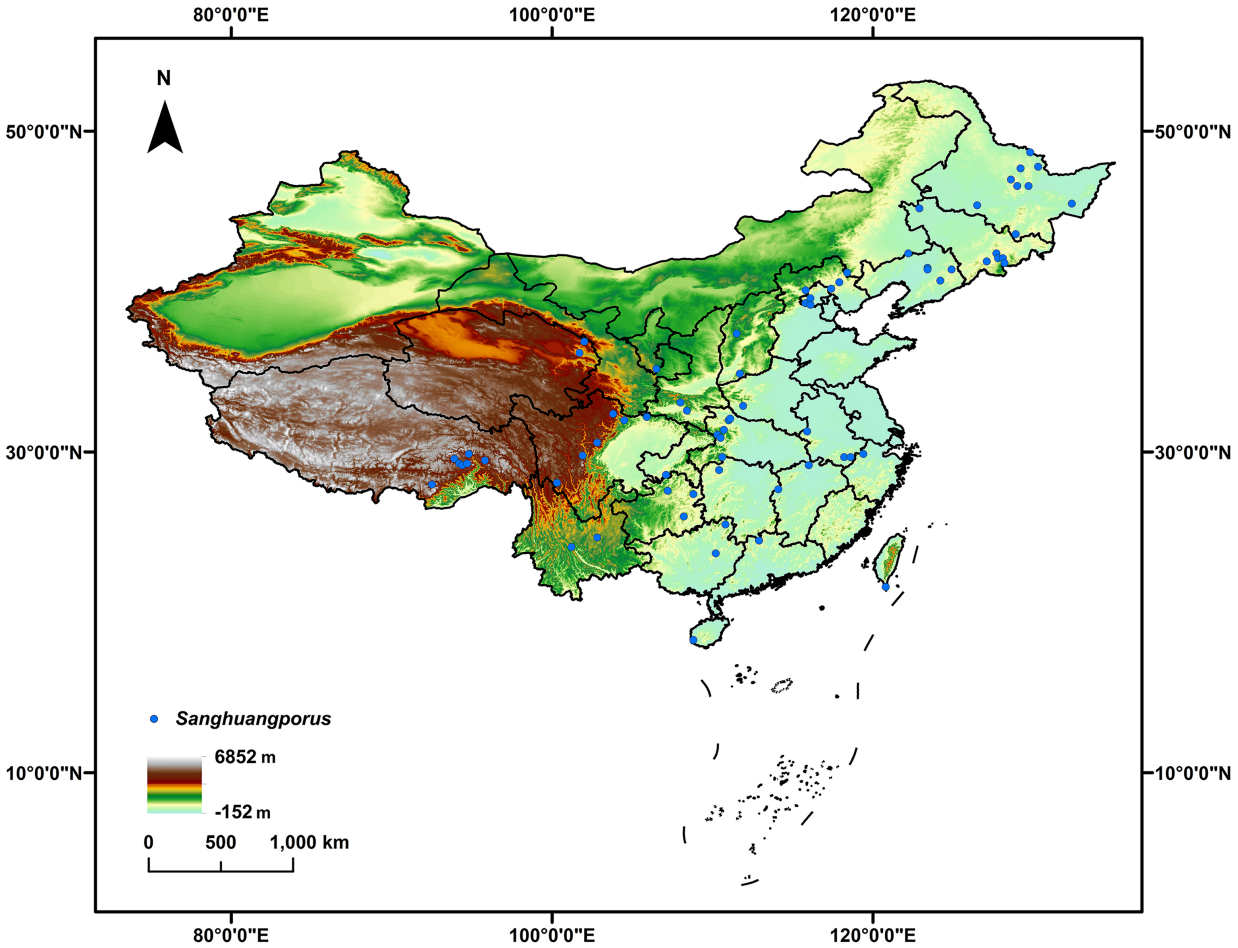
The known occurrence records of *Sanghuangporus* (blue circle) used for modeling the geographic distribution of this fungal genus in China.

Information of *Sanghuangporus*, including both published (e.g., Tian et al., 2013; Zhou et al., 2016; Shen et al., 2021) and unpublished Chinese records, was mainly retrieved from the three largest fungaria for wood-inhabiting macrofungi, *viz.* HMAS, BJFC and IFP. The abbreviations of fungaria follow Index Herbariorum.^1^ Additional information was taken from the taxonomic literature of Dr. Sheng-Hua Wu and his colleagues (Zhou et al., 2016; Wu et al., 2019b, 2020). The species identity of these records was determined preferentially based on the ITS barcoding gene (Shen et al., 2021). When ITS gene sequences were unavailable for certain records, other gene sequences and morphological characters were used to determine the species identity at least to the genus level. A total of 260 records of *Sanghuangporus* were identified. The geo-coordinates of these fungal records for modeling geographic distribution either came from field labels or were determined according to the sampling locations *via* Google Earth. All records and related ecological information are summarized in Supplementary Table S1. To avoid data redundancy of spatial autocorrelation, sampling locations less than 10 km were considered to be replications and thus deleted. Eventually, 72 records were retained for modeling the geographic distribution of *Sanghuangporus* (Figure 1).

### Environmental variables

A total of 19 bioclimatic indicators and corresponding elevation data (Table 1) were downloaded from WorldClim version 2.1 database^2^. These environmental variables from the climate data for 1970–2000 at a spatial resolution of 30″ (approximately 1 km^2^; Fick and Hijmans, 2017) were used for modeling the current geographic distribution of *Sanghuangporus*.

**Table 1:** Environmental variables used for modeling the current distribution of *Sanghuangporus* and their contributions to the predicted model.

Variable	Description	Unit	Contribution (%)	
Bio1	Annual mean temperature	°C	12.7	22.4
Bio2	Mean diurnal range	°C	–	–
Bio3	Isothermality (Bio2/Bio7) (×100)	%	3.5	2.7
Bio4	Temperature seasonality (standard deviation ×100)	°C	–	–
Bio5	Max temperature of warmest month	°C	–	–
Bio6	Min temperature of coldest month	°C	–	–
Bio7	Temperature annual range (Bio5-Bio6)	°C	5.2	5.4
Bio8	Mean temperature of wettest quarter	°C	–	–
Bio9	Mean temperature of driest quarter	°C	–	–
Bio10	Mean temperature of warmest quarter	°C	–	–
Bio11	Mean temperature of coldest quarter	°C	–	–
Bio12	Annual precipitation	mm	48.0	–
Bio13	Precipitation of wettest month	mm	–	–
Bio14	Precipitation of driest month	mm	–	–
Bio15	Precipitation seasonality (coefficient of variation)	%	3.9	3.5
Bio16	Precipitation of wettest quarter	mm	–	–
Bio17	Precipitation of driest quarter	mm	–	–
Bio18	Precipitation of warmest quarter	mm	–	57.1
Bio19	Precipitation of coldest quarter	mm	–	–
Elev	Elevation	m	9.9	8.8
Host plant	Host plant	tree/km^2^	16.9	~

The dash (−) means that the environmental variable is not included for modeling the geographic distribution due to auto correlation. The tilde (~) means that this variable is excluded for modeling.

Regarding the future scenarios, four 20-year periods, *viz.* 2030s (2021–2040), 2050s (2041–2060), 2070s (2061–2080), and 2090s (2081–2,100), each corresponding to four Shared Socio-economic Pathways (SSPs) in CMIP6 model of IPCC AR6 (Eyring et al., 2016), *viz.* SSP1-2.6, SSP2-4.5, SSP3-7.0, and SSP5-8.5 were considered. With WorldClim version 2.1 database as baseline climate, SSPs span five different shift extents of the world, *viz.* SSP1 corresponding to Sustainability—Taking the Green Road (Low challenges to mitigation and adaptation), SSP2 to Middle of the Road (Medium challenges to mitigation and adaptation), SSP3 to Regional Rivalry—A Rocky Road (High challenges to mitigation and adaptation), SSP4 to Inequality—A Road Divided (Low challenges to mitigation, high challenges to adaptation) and SSP5 to Fossil-fueled Development—Taking the Highway (High challenges to mitigation, low challenges to adaptation; see Riahi et al. (2017) for details). The future bioclimatic indicators under BCC-CSM2-MR general circulation model at a spatial resolution of 30″ (approximately 1 km^2^)^3^ were downloaded for modeling the future geographic distribution of *Sanghuangporus*. It is noteworthy that the bioclimatic indicators corresponding to SSP4 are unavailable.

Besides these commonly used bioclimatic indicators and elevation data, the host plant has been recognized as one of the most important factors restricting the growth of *Sanghuangporus* (Wu et al., 2020; Shen et al., 2021; Zhou et al., 2022). Therefore, the distribution of each host plant genus of *Sanghuangporus* (Supplementary Table S1) was retrieved from Global Biodiversity Information Facility^4^ as one of the variables. The number of host plants on each coordinate was converted to raster data by ArcGIS at a spatial resolution of 30″ (approximately 1 km^2^) for modeling the current geographic distribution of *Sanghuangporus*. To test the importance of host plants to the potential geographic distribution of *Sanghuangporus*, the current models were also predicted with the exclusion of host plant from the environmental variables. When modeling the future geographic distribution of *Sanghuangporus*, the variable of elevation was assumed to be unchanged over all analyzed time periods, while the distribution of their host plants (18 genera) under multiple future scenarios was separately predicted with the same method as used for *Sanghuangporus* (see below Model evaluation section for details). The predicted index of niche suitability of host plants in a percentage form was converted to raster data by ArcGIS at a spatial resolution of 30″ (approximately 1 km^2^). If more than one host plant genera were present in a single raster, the highest index of niche suitability from these genera was selected to represent this raster.

### Model evaluation

The potential distribution of *Sanghuangporus* was modeled using MaxEnt (Phillips et al., 2006). Of all known occurrence records, 75% were randomly selected as the training data, and the other 25% of the samples were used as the test set. The number of maximum iterations was set as 1,000 for convergence. The process was repeated 10 times to generate an averaged result for subsequent analyses. The jackknife method was used to judge the importance of environmental variables for potential distribution. Other parameters were set as default.

Some environmental variables may be spatially correlated with each other. To avoid over-fitting induced by multicollinearity of variables, Pearson correlation coefficient (r) analysis method was used to judge the correlation between primary environmental variables. When |r| > 0.8, two environmental variables were considered to be autocorrelated and the one with higher contribution rate was retained for further analyses (Supplementary Figure S1). Eventually, seven environmental variables including host plant were selected for modeling the current and future potential distribution of *Sanghuangporus* (Table 1). The response curves of these critical environmental variables to the distribution models were created.

The Area Under Receiver Operator Characteristic Curve (AUC) was estimated to determine the accuracy of the MaxEnt model for current geographic distribution (Phillips et al., 2017). In theory, the model is considered to perform well when the value of AUC is more than 0.8 and excellently when the value is more than 0.9 (Swets, 1988; Fielding and Bell, 1997). The index of niche suitability ranged from 0% to 100%, of which 0%–25% was considered to be unsuitable, 25%–50% to be of low suitability, 50%–75% to be moderately suitable, and 75%–100% to be highly suitable (Simonoff, 2003; Kim, 2013).

Comparing with the current potential distribution, the migration distance of mass centers (both coordinates and migration distances) and the variation of areas at different suitability level over above-mentioned four time periods under four future scenarios were calculated using ArcGIS 10.7.

## Results

The model for the current potential distribution of *Sanghuangporus* with the inclusion of host plant in the environmental variables is excellent as indicated by the values of AUC being 0.903, while that with exclusion of host plant from the environment variables performs well as indicated by the values of AUC being 0.899 (Supplementary Figure S2).

**Figure 2:**
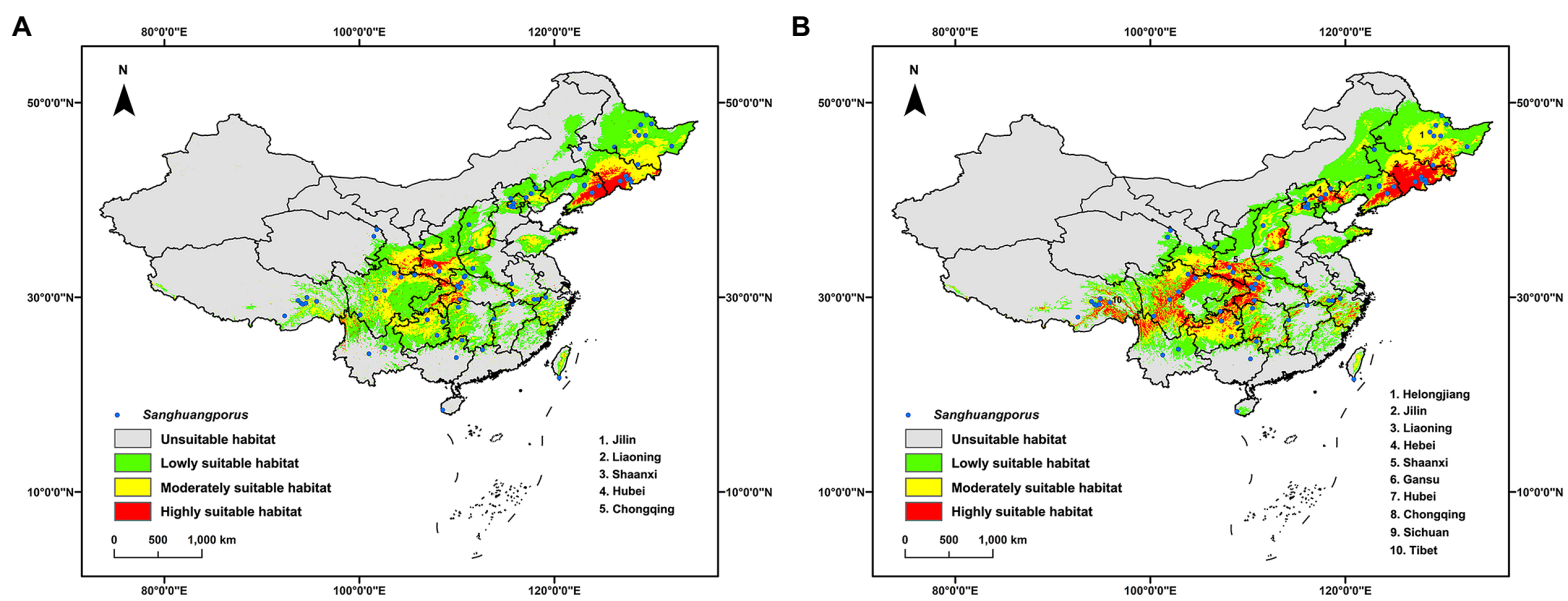
Current geographic distribution models of *Sanghuangporus* in China with the inclusion of host plant in the environmental variables **(A)** or not **(B)**. The known occurrence records are labeled as blue circle.

The current potential distribution of *Sanghuangporus* basically corresponds to the known occurrence records of *Sanghuangporus* (Figure 2A). Of the suitable habitat, the highly suitable habitat occupies 20.238 × 10^4^ km^2^ (Supplementary Table S2) and the main locations concentrate in southwestern Jilin, southeastern Liaoning, southern Shaanxi, southwestern Hubei and Chongqing (Figure 2A). In addition, many distribution spots of highly suitable habitat are scattered in northeastern, southeastern, southwestern and central China (Figure 2A). Bio1, Bio3, Bio7, Bio12, Bio15, Elev, and Host plant are the critical environmental variables to the model of the current potential distribution of *Sanghuangporus* (Table 1). Response curves of these critical environmental variables indicated that in the highly suitable habitat Bio1 ranges from-6.7 to 21.4°C, Bio3 from 16.1 to 54.0%, Bio7 from 11.5 to 63.7°C, Bio12 from 371 to 4,434 mm, Bio15 from 21 to 150%, Elev from 42 to 6,852 m, and Host plant from 1 to 1,126 tree/km^2^ (Supplementary Figure S3). Among these variables, Bio12, Host plant, and Bio1 are the most critical and contribute, respectively, 48.0%, 16.9%, and 12.7% to the model (Table 1).

When excluding host plant from the environmental variables, the potential distribution is similar to that with the inclusion of host plant (Figure 2B), but the area of the highly suitable habitat increases to 51.701 × 10^4^ km^2^ (Supplementary Table S2) and the mass center of the distribution has a migration of 261.312 km (Supplementary Table S3). Likewise, other critical environmental variables are same, but Bio18 replaces Bio12 and also contributes as the most critical variables (Table 1). It is noted that both Bio12 and Bio18 are environmental variables related to precipitation and are autocorrelated in both models (Supplementary Figure S1). In the highly suitable habitat, the ranges of critical environmental variables (Supplementary Figure S4) are also similar to those with the inclusion of host plant (Supplementary Figure S3).

**Figure 3:**
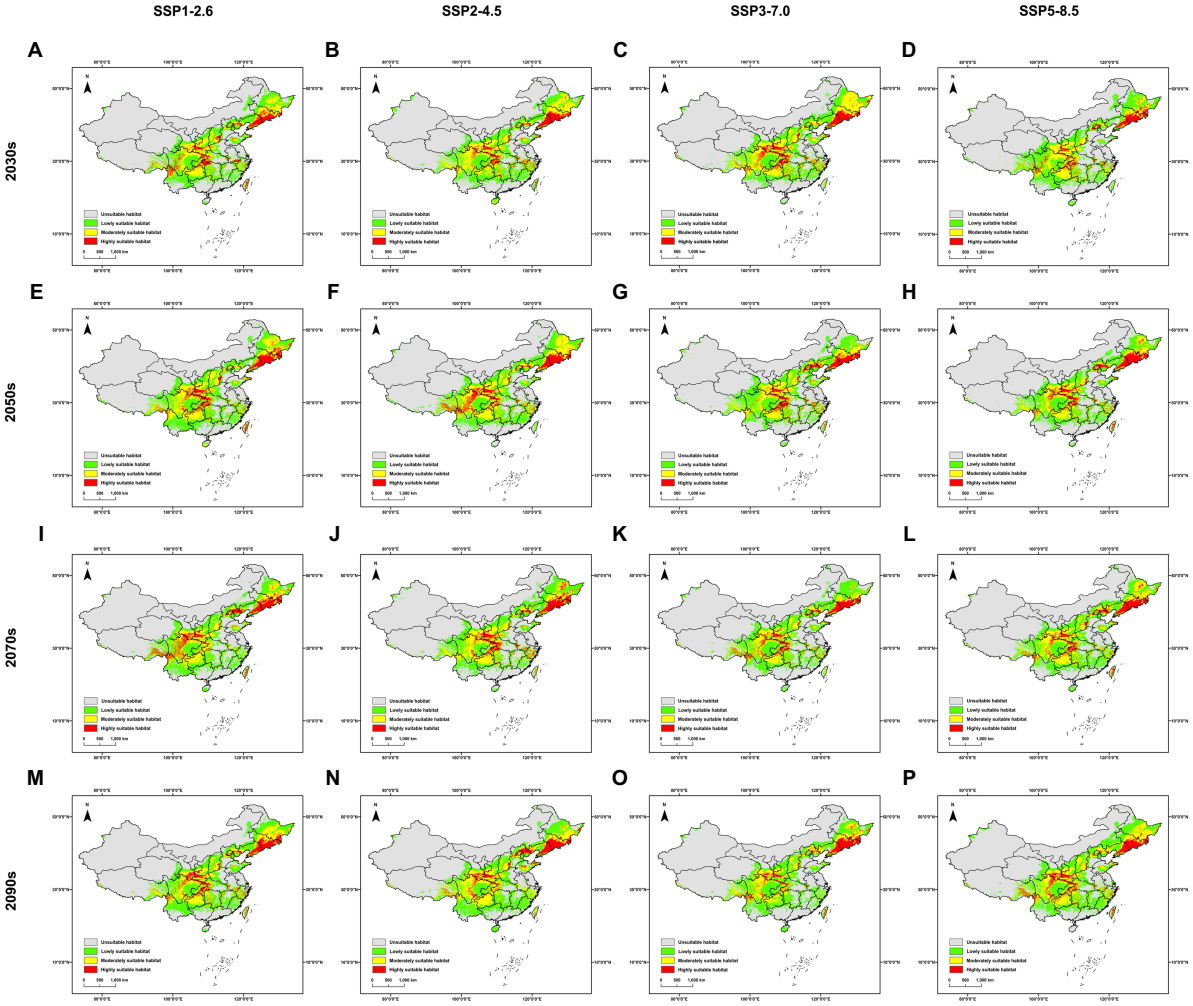
Distribution models of *Sanghuangporus* under future scenarios in China. **(A)** Under SSP1-2.6 scenario in the 2030s, **(B)** Under SSP2-4.5 scenario in the 2030s, **(C)** Under SSP3-7.0 scenario in the 2030s, **(D)** Under SSP5-8.5 scenario in the 2030s, **(E)** Under SSP1-2.6 scenario in the 2050s, **(F)** Under SSP2-4.5 scenario in the 2050s, **(G)** Under SSP3-7.0 scenario in the 2050s, **(H)** Under SSP5-8.5 scenario in the 2050s, **(I)** Under SSP1-2.6 scenario o in the 2070s, **(J)** Under SSP2-4.5 scenario in the 2070s, **(K)** Under SSP3-7.0 scenario in the 2070s, **(L)** Under SSP5-8.5 scenario in the 2070s, **(M)** Under SSP1-2.6 scenario in the 2090s, **(N)** Under SSP2-4.5 scenario in the 2090s, **(O)** Under SSP3-7.0 scenario in the 2090s, **(P)** Under SSP5-8.5 scenario in the 2090s.

Comparing with the current potential distribution, the locations of geographic distribution under future scenarios do not change much (Figure 3). Regarding the highly suitable habitat, the increase of area ranges from 67.179% under SSP5-8.5 scenario in the 2030s to 118.255% under SSP2-4.5 scenario in the 2050s, while under all four scenarios will this area increase over all four future time periods (Supplementary Table S2). The mass center of the distribution migrates mainly from 27.892 km under SSP3-7.0 scenario in the 2070s to 227.195 km under SSP2-4.5 scenario in the 2050s, except the extreme distance of 334.202 km under SSP3-7.0 scenario in the 2090s (Supplementary Table S3).

## Discussion

*Sanghuangporus* is a genus of notable edible and medicinal macrofungi (Zhou et al., 2016, 2022) and knowing where to find these species in the wild is very important for the utilization and conservation of this resource. In this study, the potential geographic distribution of *Sanghuangporus* is modeled in China. Generally, the current potential distribution corresponds to the known occurrence records of *Sanghuangporus*, and moreover provides clues to new suitable habitats (Figure 2). Therefore, future field surveys for *Sanghuangporus* should pay attention to these new habitats, especially the highly suitable habitat. This will determine whether the current modeling is accurate and generate additional occurrence records for a new round of modeling.

The current potential distribution model of *Sanghuangporus* is influenced mainly by annual precipitation and annual mean temperature (Table 1). These two environmental variables are generally important for forming fruitbodies of macrofungi from mycelia in theory (Büntgen et al., 2012; Boddy et al., 2014). Besides these two environmental variables, it is summarized that host information is also a critical variable to the distribution model of fungi that interact with hosts (Hao et al., 2020). Host plant is thus expected to be a major environmental variable to the distribution of *Sanghuangporus*, because species in this fungal genus all have a strong or weak host specificity (Wu et al., 2020; Shen et al., 2021; Zhou et al., 2022). Although host plant does not contribute a lot to the current distribution model (Table 1), its decisive role is to restrict the distribution of *Sanghuangporus* to the locations with specific trees at the genus level, even only a single tree. Moreover, when excluding host plant from environmental variables, the distribution area of the highly suitable habitat increases by 155.468% (Supplementary Table S2). This further confirms the decisive role of host plant to distribution of *Sanghuangporus*. It is noteworthy that host plant information of the current occurrence records of *Sanghuangporus* is far from comprehensive (Supplementary Table S1). For example, from all 260 records of *Sanghuangporus*, 40 are labeled on angiosperm and 49 have no host information. These occurrence records (labeled on angiosperm and no host) cannot provide host plant information for modeling potential geographic distribution. If the information of host plants is improved, either new host tree genera or known host tree genera in new locations, the modeled suitable habitat for *Sanghuangporus* will be accordingly enlarged and approach the reality. Therefore, accurate records of host plants in future field surveys may help in modeling the geographic distribution of *Sanghuangporus* and clarify the contributions of host plant to the model. Besides, the host information of *Sanghuangporus* under future scenarios is predicted (Supplementary Figure S5) from the current incomprehensive knowledge of fungal host records (Supplementary Table S1). Ideally, the distribution of the host plants related to *Sanghuangporus* will be comprehensively known under both the current and future scenarios, and then this distribution information should be accordingly set as the variable for modeling the distribution of *Sanghuangporus* under future scenarios.

Under any kind of future scenarios, human activity is not considered to be a variable for modeling. In the case that the location and mass center of *Sanghuangporus* do not change a lot under future scenarios (Supplementary Table S3), hopefully Natural Resource Conservation Areas special to *Sanghuangporus* and related protection laws could be proposed to avoid excessive collecting in the field. This strategy is consistent with the Chinese direction to post-2020 global biodiversity conservation (Wei, 2021).

Although *Sanghuangporus* is mainly utilized in China and adjacent countries, European scientists have also worked on the medicinal metabolites from unnamed species of *Sanghuangporus* from Africa (Chepkirui et al., 2018; Cheng et al., 2019). Therefore, it seems necessary for modeling the geographic distribution of *Sanghuangporus* all over the world as well as China. However, the public occurrence records of *Sanghuangporus* outside China are rarely known in East Asia (Wu et al., 2012), Vietnam (Wu et al., 2022), Central Asia (Gafforov et al., 2020), Iran (Ghobad-Nejhad, 2015), Central Europe (Tomšovský, 2015), North America (Shen et al., 2021), Australia (Wu et al., 2022) and Africa (Zhou et al., 2016). These few records are not enough to accurately perform a global distribution modeling. The accumulation of more knowledge worldwide will be helpful for the comprehensive utilization of *Sanghuangporus*.

## Data availability statement

The original contributions presented in the study are included in the article/Supplementary material, further inquiries can be directed to the corresponding author.

## Author contributions

J-HC: data curation, investigation, visualization, writing–original draft preparation. SS: data curation, investigation. L-WZ: conceptualization, investigation, writing–original draft preparation, writing–reviewing and editing. All authors contributed to the article and approved the submitted version.

## Funding

The research was financed by the National Key Research and Development Program of China (no. 2022YFC2601203), the National Natural Science Foundation of China (no. 31970012) and Biological Resources Program, Chinese Academy of Sciences (no. KFJ-BRP-017-12).

## Conflict of interest

The authors declare that the research was conducted in the absence of any commercial or financial relationships that could be construed as a potential conflict of interest.

## Publisher’s note

All claims expressed in this article are solely those of the authors and do not necessarily represent those of their affiliated organizations, or those of the publisher, the editors and the reviewers. Any product that may be evaluated in this article, or claim that may be made by its manufacturer, is not guaranteed or endorsed by the publisher.

## Footnotes

^1^ http://sweetgum.nybg.org/science/ih/

^2^ https://www.worldclim.org/data/worldclim21.html

^3^ https://www.worldclim.org/data/cmip6/cmip6_clim30s.html

^4^ https://www.gbif.org/occurrence/download/
