# Supplementary material for: Modeling current geographic distribution and future range shifts of Sanghuangporus under multiple climate change scenarios in China
Source: Front Microbiol. 2022 Dec 1;13:1064451. doi: 10.3389/fmicb.2022.1064451 (PMC9751338; doi:10.3389/fmicb.2022.1064451)
Supplement: Supplementary file 1 [file Table_2.DOCX]

Table S1 Records of *Sanghuangporus* used for modeling geographic distribution

| Species | Sampling location | Longitude | Latitude | Voucher No. | Sequence^#^ | Host plant |
| --- | --- | --- | --- | --- | --- | --- |
| *S. alpinus* | Qinghai, Haibei, Menyuan, Xianmi National Forest Park | 102°0′00″ | 37°18′00″ | Yuan 6405 (IFP) | MT348578 | *Lonicera* |
| *S. alpinus* | Qinghai, Haibei, Menyuan, Xianmi National Forest Park | 102°0′00″ | 37°18′00″ | Yuan 6413 (IFP) | & | *Lonicera* |
| *S. alpinus* | Qinghai, Haibei, Menyuan, Xianmi National Forest Park | 102°0′00″ | 37°18′00″ | Yuan 6438 (IFP) | MT343579 | angiosperm |
| *S. alpinus* | Qinghai, Xining, Xining Botanical Garden | 101°42′00″ | 36°36′00″ | Yuan 6383 (IFP) | ^ | *Syringa* |
| *S. alpinus* | Qinghai, Xining, Xining Botanical Garden | 101°42′00″ | 36°36′00″ | Yuan 6396 (IFP) | MT348577 | *Lonicera* |
| *S. alpinus* | Sichuan, Aba, Songpan, Huanglong Scenic Spot | 103°48′00″ | 32°45′00″ | Cui 10651 (BJFC) | & | *Lonicera* |
| *S. alpinus* | Sichuan, Aba, Songpan, Huanglong Scenic Spot | 103°48′00″ | 32°45′00″ | Cui 10654 (BJFC) | ^ | *Lonicera* |
| *S. alpinus* | Sichuan, Aba, Songpan, Huanglong Scenic Spot | 103°48′00″ | 32°45′00″ | Cui 10671 (BJFC) | & | *Lonicera* |
| *S. alpinus* | Sichuan, Aba, Songpan, Huanglong Scenic Spot | 103°48′00″ | 32°45′00″ | Dai 4233 (BJFC) | ^ | *Lonicera* |
| *S. alpinus* | Sichuan, Aba, Xiaojin, Siguniang Mountain | 102°48′00″ | 31°0′00″ | Cui 10690 (BJFC) | ^ | *Lonicera* |
| *S. alpinus* | Sichuan, Aba, Xiaojin, Siguniang Mountain | 102°48′00″ | 31°0′00″ | Cui 10691 (BJFC) | & | *Lonicera* |
| *S. alpinus* | Sichuan, Aba, Xiaojin, Siguniang Mountain | 102°48′00″ | 31°0′00″ | Cui 10692 (BJFC) | ^ | *Lonicera* |
| *S. alpinus* | Sichuan, Aba, Xiaojin, Siguniang Mountain | 102°48′00″ | 31°0′00″ | Cui 10706 (BJFC) | ^ | *Lonicera* |
| *S. alpinus* | Sichuan, Aba, Xiaojin, Siguniang Mountain | 102°48′00″ | 31°0′00″ | Cui 10708 (BJFC) | & | *Lonicera* |
| *S. alpinus* | Sichuan, Aba, Xiaojin, Siguniang Mountain | 102°48′00″ | 31°0′00″ | Cui 10711 (BJFC) | & | *Lonicera* |
| *S. alpinus* | Sichuan, Garzê, Dacheng, Yading National Nature Reserve | 100°20′57″ | 28°27′33″ | Cui 12474 (BJFC) | MF772783 | *Lonicera* |
| *S. alpinus* | Sichuan, Garzê, Dacheng, Yading National Nature Reserve | 100°20′57″ | 28°27′33″ | Cui 12485 (BJFC) | MF772781 | *Lonicera* |
| *S. alpinus* | Sichuan, Garzê, Kangding, Mugecuo | 101°54′26″ | 30°11′43″ | Cui 12444 (BJFC) | MF772782 | *Lonicera* |
| *S. alpinus* | Tibet, Linzhi | 94°12′00″ | 29°42′00″ | Cui 9393 (BJFC) | ^ | *Lonicera* |
| *S. alpinus* | Tibet, Linzhi | 94°12′00″ | 29°42′00″ | Cui 9396 (BJFC) | ^ |  |
| *S. alpinus* | Tibet, Linzhi | 94°12′00″ | 29°42′00″ | Cui 9400 (BJFC) | & |  |
| *S. alpinus* | Tibet, Linzhi | 94°12′00″ | 29°42′00″ | Cui 9403 (BJFC) | & |  |
| *S. alpinus* | Tibet, Linzhi | 94°12′00″ | 29°42′00″ | Cui 9407 (BJFC) | & |  |
| *S. alpinus* | Tibet, Linzhi | 94°12′00″ | 29°42′00″ | Cui 9411 (BJFC) | & |  |
| *S. alpinus* | Tibet, Linzhi | 94°12′00″ | 29°42′00″ | Cui 9421 (BJFC) | & |  |
| *S. alpinus* | Tibet, Linzhi | 94°12′00″ | 29°42′00″ | Cui 9426 (BJFC) | & |  |
| *S. alpinus* | Tibet, Linzhi | 94°12′00″ | 29°42′00″ | Cui 9432 (BJFC) | & |  |
| *S. alpinus* | Tibet, Linzhi | 94°12′00″ | 29°42′00″ | Cui 9439 (BJFC) | & |  |
| *S. alpinus* | Tibet, Linzhi | 94°12′00″ | 29°42′00″ | Cui 9441 (BJFC) | & |  |
| *S. alpinus* | Tibet, Linzhi | 94°12′00″ | 29°42′00″ | Cui 9446 (BJFC) | & |  |
| *S. alpinus* | Tibet, Linzhi | 94°12′00″ | 29°42′00″ | Cui 9449 (BJFC) | ^ |  |
| *S. alpinus* | Tibet, Linzhi | 94°12′00″ | 29°42′00″ | Cui 9452 (BJFC) | ^ |  |
| *S. alpinus* | Tibet, Linzhi | 94°12′00″ | 29°42′00″ | HMAS 265347 | ^ |  |
| *S. alpinus* | Tibet, Linzhi, Bayi | 94°24′00″ | 29°36′00″ | Dai 5630 (IFP) | ^ | *Lonicera* |
| *S. alpinus* | Tibet, Linzhi, Bayi | 94°24′00″ | 29°36′00″ | Dai 5650 (IFP) | ^ | *Lonicera* |
| *S. alpinus* | Tibet, Linzhi, Bayi | 94°24′00″ | 29°36′00″ | Yu 35 (BJFC) | JQ860312 | *Lonicera* |
| *S. alpinus* | Tibet, Linzhi, Bomi | 95°46′15″ | 29°51′33″ | Cui 9463 (BJFC) | ^ |  |
| *S. alpinus* | Tibet, Linzhi, Bomi | 95°46′15″ | 29°51′33″ | Cui 9477 (BJFC) | ^ |  |
| *S. alpinus* | Tibet, Linzhi, Bomi | 95°46′15″ | 29°51′33″ | Cui 9484 (BJFC) | ^ |  |
| *S. alpinus* | Tibet, Linzhi, Bomi | 95°46′15″ | 29°51′33″ | Cui 9500 (BJFC) | ^ |  |
| *S. alpinus* | Tibet, Linzhi, Bomi | 95°46′15″ | 29°51′33″ | HMAS 265369 | ^ |  |
| *S. alpinus* | Tibet, Linzhi, Bomi, Yigong | 94°48′00″ | 30°18′00″ | Cui 10977 (BJFC) | & |  |
| *S. alpinus* | Tibet, Linzhi, Gongbujiangda, Cuogao | 93°54′00″ | 30°0′00″ | Yu 231 (IFP) | ^ | *Lonicera* |
| *S. alpinus* | Tibet, Linzhi, Kadinggou Park | 94°12′00″ | 29°42′00″ | Cui 12308 (BJFC) | & | *Quercus* |
| *S. alpinus* | Tibet, Linzhi, Lulang | 94°42′00″ | 29°42′00″ | Cui 9343 (BJFC) | ^ | *Lonicera* |
| *S. alpinus* | Tibet, Linzhi, Lulang | 94°42′00″ | 29°42′00″ | Cui 9387 (BJFC) | ^ |  |
| *S. alpinus* | Tibet, Linzhi, Lulang | 94°42′00″ | 29°42′00″ | Cui 9390 (BJFC) | ^ |  |
| *S. alpinus* | Tibet, Linzhi, Lulang | 94°42′00″ | 29°42′00″ | Cui 9646 (BJFC) | JQ860313 | angiosperm |
| *S. alpinus* | Tibet, Linzhi, Lulang | 94°42′00″ | 29°42′00″ | Cui 9652 (BJFC) | JQ860309 | angiosperm |
| *S. alpinus* | Tibet, Linzhi, Lulang | 94°42′00″ | 29°42′00″ | Cui 9658 (BJFC) | JQ860310 | angiosperm |
| *S. alpinus* | Tibet, Linzhi, Lulang | 94°42′00″ | 29°42′00″ | Cui 9666 (BJFC) | JQ860311 | angiosperm |
| *S. alpinus* | Tibet, Linzhi, Lulang, Sejila Mountain | 94°42′00″ | 29°42′00″ | Cui 9675 (BJFC) | ^ |  |
| *S. alpinus* | Tibet, Linzhi, Lulang, Sejila Mountain | 94°42′00″ | 29°42′00″ | Cui 9686 (BJFC) | & |  |
| *S. alpinus* | Tibet, Linzhi, Lulang, Sejila Mountain | 94°42′00″ | 29°42′00″ | Cui 12123 (BJFC) | & | *Lonicera* |
| *S. alpinus* | Tibet, Linzhi, Lulang, Sejila Mountain | 94°42′00″ | 29°42′00″ | Cui 12138 (BJFC) | & | *Lonicera* |
| *S. alpinus* | Tibet, Linzhi, Lulang, Sejila Mountain | 94°42′00″ | 29°42′00″ | Cui 12186 (BJFC) | & | *Lonicera* |
| *S. alpinus* | Tibet, Linzhi, Lulang, Sejila Mountain | 94°42′00″ | 29°42′00″ | LWZ 20140917-3 (IFP) | ^ | *Lonicera* |
| *S. alpinus* | Tibet, Linzhi, Lulang, Sejila Mountain | 94°42′00″ | 29°42′00″ | LWZ 20140917-5 (IFP) | ^ | *Lonicera* |
| *S. alpinus* | Tibet, Linzhi, Lulang, Sejila Mountain | 94°42′00″ | 29°42′00″ | LWZ 20140917-7 (IFP) | ^ | *Lonicera* |
| *S. alpinus* | Tibet, Linzhi, Lulang, Sejila Mountain | 94°42′00″ | 29°42′00″ | LWZ 20140917-8 (IFP) | ^ | *Lonicera* |
| *S. alpinus* | Tibet, Linzhi, Lulang, Sejila Mountain | 94°42′00″ | 29°42′00″ | LWZ 20140918-10 (IFP) | ^ | *Lonicera* |
| *S. alpinus* | Tibet, Shannan, Longzi | 92°27′41″ | 28°24′29″ | HMAS 274144 | ^ |  |
| *S. baumii* | Heilongjiang, Harbin, Zhaolin Park | 126°30′00″ | 45°48′00″ | Dai 16897 (BJFC) | & | *Syringa* |
| *S. baumii* | Heilongjiang, Harbin, Zhaolin Park | 126°30′00″ | 45°48′00″ | Dai 16898 (BJFC) | & | *Syringa* |
| *S. baumii* | Heilongjiang, Harbin, Zhaolin Park | 126°30′00″ | 45°48′00″ | Dai 16900 (BJFC) | MF772785 | *Syringa* |
| *S. baumii* | Heilongjiang, Harbin, Zhaolin Park | 126°30′00″ | 45°48′00″ | Dai 16902 (BJFC) | & | *Syringa* |
| *S. baumii* | Heilongjiang, Hegang, Luobei, Hebei Forestry Bureau United Forest Farm | 130°18′00″ | 48°12′00″ | Yuan 5115 (IFP) | ^ | *Syringa* |
| *S. baumii* | Heilongjiang, Jiamusi, Tangyuan, Daliangzihe National Forest Park | 129°42′00″ | 47°0′00″ | Yuan 4909 (IFP) | KY328310 | angiosperm |
| *S. baumii* | Heilongjiang, Jiamusi, Tangyuan, Daliangzihe National Forest Park | 129°42′00″ | 47°0′00″ | Yuan 4929 (IFP) | KY328306 | *Alnus* |
| *S. baumii* | Heilongjiang, Jixi, Hulin, Seven Tiger Forest Farm | 132°24′00″ | 45°54′00″ | Yuan 436 (IFP) | ^ | *Alnus* |
| *S. baumii* | Heilongjiang, Yichun | 128°33′36″ | 47°25′12″ | HMAS 253613 | ^ |  |
| *S. baumii* | Heilongjiang, Yichun | 128°33′36″ | 47°25′12″ | HMAS 272777 | ^ |  |
| *S. baumii* | Heilongjiang, Yichun, Dailing, Liangshui National Nature Reserve | 129°0′00″ | 47°0′00″ | Cui 11537 (BJFC) | & | *Syringa* |
| *S. baumii* | Heilongjiang, Yichun, Dailing, Liangshui National Nature Reserve | 129°0′00″ | 47°0′00″ | Cui 11538 (BJFC) | & | *Syringa* |
| *S. baumii* | Heilongjiang, Yichun, Dailing, Liangshui National Nature Reserve | 129°0′00″ | 47°0′00″ | Cui 11539 (BJFC) | & | *Syringa* |
| *S. baumii* | Heilongjiang, Yichun, Dailing, Liangshui National Nature Reserve | 129°0′00″ | 47°0′00″ | Cui 11540 (BJFC) | ^ | *Syringa* |
| *S. baumii* | Heilongjiang, Yichun, Dailing, Liangshui National Nature Reserve | 129°0′00″ | 47°0′00″ | Cui 11541 (BJFC) | & | *Syringa* |
| *S. baumii* | Heilongjiang, Yichun, Dailing, Liangshui National Nature Reserve | 129°0′00″ | 47°0′00″ | Yuan 5218 (IFP) | ^ | *Alnus* |
| *S. baumii* | Heilongjiang, Yichun, Dailing, Liangshui National Nature Reserve | 129°0′00″ | 47°0′00″ | Yuan 5255 (IFP) | ^ | *Syringa* |
| *S. baumii* | Heilongjiang, Yichun, Dailing, Liangshui National Nature Reserve | 129°0′00″ | 47°0′00″ | Yuan 5303 (IFP) | ^ | angiosperm |
| *S. baumii* | Heilongjiang, Yichun, Jiayin, Maolangou National Forest Park | 129°48′00″ | 49°6′00″ | Cui 11903 (BJFC) | KY328305 | *Alnus* |
| *S. baumii* | Heilongjiang, Yichun, Jiayin, Maolangou National Forest Park | 129°48′00″ | 49°6′00″ | Cui 11904 (BJFC) | & | *Alnus* |
| *S. baumii* | Heilongjiang, Yichun, Jiayin, Maolangou National Forest Park | 129°48′00″ | 49°6′00″ | Cui 11905 (BJFC) | ^ | *Alnus* |
| *S. baumii* | Heilongjiang, Yichun, Jiayin, Maolangou National Forest Park | 129°48′00″ | 49°6′00″ | Cui 11907 (BJFC) | & | *Alnus* |
| *S. baumii* | Heilongjiang, Yichun, Jiayin, Maolangou National Forest Park | 129°48′00″ | 49°6′00″ | Dai 18460 (BJFC) | ^ | *Alnus* |
| *S. baumii* | Heilongjiang, Yichun, Wuying, Fenglin National Nature Reserve | 129°12′00″ | 48°6′00″ | Cui 9843 (BJFC) | & |  |
| *S. baumii* | Heilongjiang, Yichun, Wuying, Fenglin National Nature Reserve | 129°12′00″ | 48°6′00″ | Cui 9845 (BJFC) | & | *Syringa* |
| *S. baumii* | Heilongjiang, Yichun, Wuying, Fenglin National Nature Reserve | 129°12′00″ | 48°6′00″ | Cui 9876 (BJFC) | & | *Betula* |
| *S. baumii* | Heilongjiang, Yichun, Wuying, Fenglin National Nature Reserve | 129°12′00″ | 48°6′00″ | Cui 9891 (BJFC) | ^ |  |
| *S. baumii* | Heilongjiang, Yichun, Wuying, Fenglin National Nature Reserve | 129°12′00″ | 48°6′00″ | Cui 9916 (BJFC) | & | *Prunus* |
| *S. baumii* | Heilongjiang, Yichun, Wuying, Fenglin National Nature Reserve | 129°12′00″ | 48°6′00″ | Cui 9919 (IFP) | ^ | *Acer* |
| *S. baumii* | Heilongjiang, Yichun, Wuying, Fenglin National Nature Reserve | 129°12′00″ | 48°6′00″ | Cui 11765 (BJFC) | ^ |  |
| *S. baumii* | Heilongjiang, Yichun, Wuying, Fenglin National Nature Reserve | 129°12′00″ | 48°6′00″ | Cui 11766 (BJFC) | & |  |
| *S. baumii* | Heilongjiang, Yichun, Wuying, Fenglin National Nature Reserve | 129°12′00″ | 48°6′00″ | Cui 11767 (BJFC) | & |  |
| *S. baumii* | Heilongjiang, Yichun, Wuying, Fenglin National Nature Reserve | 129°12′00″ | 48°6′00″ | Cui 11768 (BJFC) | & |  |
| *S. baumii* | Heilongjiang, Yichun, Wuying, Fenglin National Nature Reserve | 129°12′00″ | 48°6′00″ | Cui 11769 (BJFC) | MF772784 | angiosperm |
| *S. baumii* | Heilongjiang, Yichun, Wuying, Fenglin National Nature Reserve | 129°12′00″ | 48°6′00″ | Dai 3645 (IFP) | ^ | *Syringa* |
| *S. baumii* | Heilongjiang, Yichun, Wuying, Fenglin National Nature Reserve | 129°12′00″ | 48°6′00″ | Dai 3656 (IFP) | ^ | *Syringa* |
| *S. baumii* | Heilongjiang, Yichun, Wuying, Fenglin National Nature Reserve | 129°12′00″ | 48°6′00″ | Dai 3674 (IFP) | ^ | *Syringa* |
| *S. baumii* | Heilongjiang, Yichun, Wuying, Fenglin National Nature Reserve | 129°12′09″ | 48°8′20″ | Dai 3683 (IFP) | JN642567 | *Syringa* |
| *S. baumii* | Heilongjiang, Yichun, Wuying, Fenglin National Nature Reserve | 129°12′09″ | 48°8′20″ | Dai 3684 (IFP) | JN642568 | *Syringa* |
| *S. baumii* | Heilongjiang, Yichun, Wuying, Fenglin National Nature Reserve | 129°12′09″ | 48°8′20″ | Dai 3694 (IFP) | JN642569 | *Syringa* |
| *S. baumii* | Jilin, Yanbian, Antu, Baoma | 128°6′00″ | 42°30′00″ | Dai 1993 (IFP) | ^ | *Syringa* |
| *S. baumii* | Jilin, Yanbian, Antu, Changbai Mountain National Nature Reserve | 128°12′00″ | 42°12′00″ | Dai 3762 (BJFC) | ^ | *Acer* |
| *S. baumii* | Jilin, Yanbian, Antu, Changbai Mountain National Nature Reserve | 128°12′00″ | 42°12′00″ | Wei 3203 (IFP) | ^ | *Syringa* |
| *S. lonicericola* | Jilin, Jilin, Huadian, Jiapigou, Dongxing | 127°42′00″ | 42°48′00″ | Dai 1611 (IFP) | ^ | *Lonicera* |
| *S. lonicericola* | Heilongjiang, Mudanjiang, Ning'an, Jingbohu National Scenic Spot | 128°54′00″ | 44°0′00″ | Dai 8311 (IFP) | ^ | *Lonicera* |
| *S. lonicericola* | Heilongjiang, Mudanjiang, Ning'an, Jingbohu National Scenic Spot | 128°54′00″ | 44°0′00″ | Dai 8322 (IFP) | JN642571 | *Lonicera* |
| *S. lonicericola* | Heilongjiang, Mudanjiang, Ning'an, Jingbohu National Scenic Spot | 128°54′00″ | 44°0′00″ | Dai 8335 (IFP) | JN642573 | *Lonicera* |
| *S. lonicericola* | Heilongjiang, Mudanjiang, Ning'an, Jingbohu National Scenic Spot | 128°54′00″ | 44°0′00″ | Dai 8336 (IFP) | & | *Lonicera* |
| *S. lonicericola* | Heilongjiang, Mudanjiang, Ning'an, Jingbohu National Scenic Spot | 128°54′00″ | 44°0′00″ | Dai 8340 (BJFC) | JN642574 | *Lonicera* |
| *S. lonicericola* | Heilongjiang, Mudanjiang, Ning'an, Jingbohu National Scenic Spot | 128°54′00″ | 44°0′00″ | Dai 8376 (BJFC) | JQ860308 | *Lonicera* |
| *S. lonicericola* | Heilongjiang, Mudanjiang, Ning'an, Jingbohu National Scenic Spot | 128°54′00″ | 44°0′00″ | Dai 8377 (IFP) | & | *Lonicera* |
| *S. lonicericola* | Heilongjiang, Mudanjiang, Ning'an, Jingbohu National Scenic Spot | 128°54′00″ | 44°0′00″ | Dai 8978 (IFP) | ^ | angiosperm |
| *S. lonicericola* | Jilin, Baishan, Fusong, Lushuihe Forest Farm | 127°48′00″ | 42°30′00″ | Cui 10105 (BJFC) | ^ | *Lonicera* |
| *S. lonicericola* | Liaoning, Fushun, Qingyuan Forest Ecosystem Observation and Research Station | 124°54′00″ | 41°48′00″ | Dai 15991 (BJFC) | & | *Lonicera* |
| *S. lonicericola* | Liaoning, Shenyang, Beiling Park | 123°24′00″ | 41°54′00″ | Wei 1154 (IFP) | ^ | *Crataegus* |
| *S. lonicericola* | Liaoning, Shenyang, Beiling Park | 123°24′00″ | 41°54′00″ | Wei 1165 (IFP) | ^ | *Lonicera* |
| *S. lonicericola* | Liaoning, Shenyang, Beiling Park | 123°25′51″ | 41°50′42″ | Dai 17304 (BJFC) | MT348582 | *Lonicera* |
| *S. lonicericola* | Neimenggu, Tongliao, Ganqika, Daqinggou National Nature Reserve | 122°12′00″ | 42°48′00″ | Dai 3983 (BJFC) | & | *Lonicera* |
| *S. lonicericola* | Neimenggu, Tongliao, Ganqika, Daqinggou National Nature Reserve | 122°12′00″ | 42°48′00″ | Dai 3986 (IFP) | & | *Lonicera* |
| *S. lonicericola* | Neimenggu, Tongliao, Ganqika, Daqinggou National Nature Reserve | 122°12′00″ | 42°48′00″ | Dai 15814 (BJFC) | ^ | *Lonicera* |
| *S. lonicericola* | Neimenggu, Tongliao, Ganqika, Daqinggou National Nature Reserve | 122°12′00″ | 42°48′00″ | Dai 15815 (BJFC) | & | *Lonicera* |
| *S. lonicericola* | Neimenggu, Tongliao, Ganqika, Daqinggou National Nature Reserve | 122°12′00″ | 42°48′00″ | Dai 15817 (BJFC) | ^ | *Lonicera* |
| *S. quercicola* | Anhui, Lu'an, Jinzhai | 115°54′00″ | 31°42′00″ | Dai 12478 (IFP) | ^ | *Quercus* |
| *S. quercicola* | Anhui, Xuancheng, Jixi | 118°36′00″ | 30°6′00″ | Dai 10633 (BJFC) | ^ |  |
| *S. quercicola* | Chongqing, Nanchuan, Jinfo Mountain National Nature Reserve | 107°6′00″ | 29°0′00″ | Dai 13947 (BJFC) | KY328309 |  |
| *S. quercicola* | Chongqing, Nanchuan, Jinfo Mountain National Nature Reserve | 107°6′00″ | 29°0′00″ | Dai 13954 (BJFC) | & |  |
| *S. quercicola* | Henan, Nanyang, Neixiang, Baotianman National Nature Reserve | 111°54′00″ | 33°18′00″ | Li 269 (IFP) | ^ | angiosperm |
| *S. quercicola* | Henan, Nanyang, Neixiang, Baotianman National Nature Reserve | 111°54′00″ | 33°18′00″ | Li 417 (IFP) | ^ | angiosperm |
| *S. quercicola* | Henan, Nanyang, Neixiang, Baotianman National Nature Reserve | 111°54′00″ | 33°18′00″ | Li 445 (IFP) | KY328311 | angiosperm |
| *S. quercicola* | Henan, Nanyang, Neixiang, Baotianman National Nature Reserve | 111°54′00″ | 33°18′00″ | Li 454 (IFP) | ^ | angiosperm |
| *S. quercicola* | Henan, Nanyang, Neixiang, Baotianman National Nature Reserve | 111°54′00″ | 33°18′00″ | Li 1139 (IFP) | ^ | angiosperm |
| *S. quercicola* | Henan, Nanyang, Neixiang, Baotianman National Nature Reserve | 111°54′00″ | 33°18′00″ | Li 1149 (IFP) | KY328312 | *Quercus* |
| *S. quercicola* | Henan, Nanyang, Neixiang, Baotianman National Nature Reserve | 111°55′48″ | 33°18′00″ | Wei 7575 (IFP) | MT348587 | *Quercus* |
| *S. quercicola* | Hubei, Shennongjia Forestry District | 110°42′00″ | 31°45′11″ | Li 804 (IFP) | ^ | angiosperm |
| *S. quercicola* | Hubei, Shennongjia Forestry District, Jinhou Mountain | 110°18′00″ | 31°30′00″ | Dai 5875 (IFP) | ^ | *Cerasus* |
| *S. quercicola* | Hubei, Shennongjia Forestry District, Jinhou Mountain | 110°18′00″ | 31°30′00″ | Wei 1968 (IFP) | ^ | *Acer* |
| *S. quercicola* | Hubei, Shennongjia Forestry District, Jinhou Mountain | 110°18′00″ | 31°30′00″ | Wei 2010 (IFP) | ^ | angiosperm |
| *S. quercicola* | Hubei, Shennongjia Forestry District, Jinhou Mountain | 110°40′05″ | 31°45′11″ | Wu 1805-2 | MK400422 | *Toxicodendron* |
| *S. quercicola* | Hubei, Shennongjia Forestry District, Jinhou Mountain | 110°40′05″ | 31°45′11″ | Wu 1805-3 | MK400423 | *Toxicodendron* |
| *S. quercicola* | Hubei, Shennongjia Forestry District, Jinhou Mountain | 110°40′05″ | 31°45′11″ | Wu 1805-5 | MK400424 | *Toxicodendron* |
| *S. quercicola* | Hubei, Shennongjia Forestry District, Jinhou Mountain | 110°40′05″ | 31°45′11″ | Wu 1807-2 | MK729538 | *Toxicodendron* |
| *S. quercicola* | Hubei, Shennongjia Forestry District, Jinhou Mountain | 110°40′05″ | 31°45′11″ | Wu 1807-3 | MK729540 | *Toxicodendron* |
| *S. quercicola* | Hubei, Shennongjia Forestry District, Jinhou Mountain | 110°40′05″ | 31°45′11″ | Wu 1807-4 | MK729539 | *Toxicodendron* |
| *S. quercicola* | Hubei, Shiyan, Wudang Mountain | 111°6′00″ | 32°30′00″ | Li 488 (IFP) | ^ | angiosperm |
| *S. quercicola* | Hubei, Shiyan, Wudang Mountain | 111°6′00″ | 32°30′00″ | Li 496 (IFP) | ^ | angiosperm |
| *S. quercicola* | Hubei, Shiyan, Wudang Mountain, Jinding | 111°0′59″ | 32°24′17″ | LWZ 20170821-13 (IFP) | MT348584 | angiosperm |
| *S. quercicola* | Hubei, Shiyan, Wudang Mountain, Jinding | 111°0′59″ | 32°24′17″ | LWZ 20170821-14 (IFP) | MT348585 | angiosperm |
| *S. quercicola* | Hubei, Shiyan, Wudang Mountain, Jinding | 111°0′59″ | 32°24′17″ | LWZ 20170821-18 (IFP) | MT348586 | angiosperm |
| *S. sanghuang* | Jilin, Baicheng | 122°52′08″ | 45°36′32″ | Wu 0903-1 | JN794061 | *Morus* |
| *S. sanghuang* | Shanxi, Ankang, Shiquan, Chihe, Wuai | 108°21′19″ | 32°57′19″ | Cui 14419 (BJFC) | MF772789 | *Morus* |
| *S. sanghuang* | Shanxi, Ankang, Shiquan, Chihe, Wuai | 108°21′19″ | 32°57′19″ | Cui 14420 (BJFC) | MF772790 | *Morus* |
| *S. sanghuang* | Sichuan, Guanyuan, Chaotian | 105°54′00″ | 32°36′00″ | Dai 12723 (BJFC) | JQ860316 | *Morus* |
| *S. sanghuang* | Sichuan, Mianyang, Pingwu | 104°30′00″ | 32°24′00″ | Dai 18460 (BJFC) | ^ | *Morus* |
| *S. sanghuang* | Yunnan, Kunming, Heilongtan Park | 102°45′25″ | 25°8′52″ | LWZ 20180927-3 (HMAS) | MT348588 | *Morus* |
| *S. sanghuang* | Yunnan, Kunming, Heilongtan Park | 102°48′00″ | 25°6′00″ | Dai 13039 (BJFC) | & | *Morus* |
| *S. subbaumii* | Beijing, Fanshan, Shangfang Mountain National Forest Park | 115°49′14″ | 39°40′23″ | LWZ 20190722-18 (HMAS) | MT348581 | angiosperm |
| *S. subbaumii* | Beijing, Haidian, Beijing Botanical Garden | 116°6′00″ | 40°0′00″ | Cui 6797 (BJFC) | & | *Syringa* |
| *S. subbaumii* | Beijing, Haidian, Beijing Botanical Garden | 116°6′00″ | 40°0′00″ | Cui 10237 (BJFC) | & | *Syringa* |
| *S. subbaumii* | Beijing, Haidian, Beijing Botanical Garden | 116°6′00″ | 40°0′00″ | Dai 10637 (BJFC) | & | *Syringa* |
| *S. subbaumii* | Beijing, Haidian, Beijing Botanical Garden | 116°6′00″ | 40°0′00″ | Dai 10638 (BJFC) | & | *Syringa* |
| *S. subbaumii* | Beijing, Haidian, Beijing Botanical Garden | 116°6′00″ | 40°0′00″ | Dai 10639 (BJFC) | & | *Syringa* |
| *S. subbaumii* | Beijing, Haidian, Beijing Botanical Garden | 116°6′00″ | 40°0′00″ | Dai 11464 (BJFC) | ^ | *Syringa* |
| *S. subbaumii* | Beijing, Haidian, Fragrant Mountain Park | 116°6′36″ | 39°35′24″ | Wu 0910-54 | JH642570 | *Syringa* |
| *S. subbaumii* | Beijing, Yanqing, Songshan Mountain National Nature Reserve | 115°48′00″ | 40°30′00″ | Dai 6626 (IFP) | ^ | *Syringa* |
| *S. subbaumii* | Beijing, Yanqing, Songshan Mountain National Nature Reserve | 115°48′00″ | 40°30′00″ | Dai 6667 (IFP) | ^ | *Fraxinus* |
| *S. subbaumii* | Hebei, Chengde, Chengde Imperial Summer Resort | 117°54′00″ | 41°0′00″ | Dai 17133 (BJFC) | & | *Lonicera* |
| *S. subbaumii* | Hebei, Chengde, Chengde Imperial Summer Resort | 117°54′00″ | 41°0′00″ | Dai 17134 (BJFC) | & | *Lonicera* |
| *S. subbaumii* | Hebei, Chengde, Chengde Imperial Summer Resort | 117°54′00″ | 41°0′00″ | Dai 17135 (BJFC) | & | *Syringa* |
| *S. subbaumii* | Hebei, Chengde, Chengde Imperial Summer Resort | 117°54′00″ | 41°0′00″ | Dai 17136 (BJFC) | & | *Syringa* |
| *S. subbaumii* | Hebei, Chengde, Chengde Imperial Summer Resort | 117°54′00″ | 41°0′00″ | Dai 17137 (BJFC) | ^ | *Syringa* |
| *S. subbaumii* | Hebei, Chengde, Chengde Imperial Summer Resort | 117°54′00″ | 41°0′00″ | Dai 17138 (BJFC) | & | *Syringa* |
| *S. subbaumii* | Hebei, Chengde, Chengde Imperial Summer Resort | 117°54′00″ | 41°0′00″ | Dai 17139 (BJFC) | & | *Syringa* |
| *S. subbaumii* | Hebei, Chengde, Chengde Imperial Summer Resort | 117°54′00″ | 41°0′00″ | Dai 17140 (BJFC) | & | *Syringa* |
| *S. subbaumii* | Hebei, Chengde, Chengde Imperial Summer Resort | 117°54′00″ | 41°0′00″ | Dai 17141 (BJFC) | & | *Syringa* |
| *S. subbaumii* | Hebei, Chengde, Chengde Imperial Summer Resort | 117°54′00″ | 41°0′00″ | Dai 17142 (BJFC) | & | *Syringa* |
| *S. subbaumii* | Hebei, Chengde, Chengde Imperial Summer Resort | 117°54′00″ | 41°0′00″ | Dai 17143 (BJFC) | & | *Syringa* |
| *S. subbaumii* | Hebei, Chengde, Chengde Imperial Summer Resort | 117°54′00″ | 41°0′00″ | Dai 17144 (BJFC) | & | *Syringa* |
| *S. subbaumii* | Hebei, Chengde, Chengde Imperial Summer Resort | 117°54′00″ | 41°0′00″ | Dai 17145 (BJFC) | & | *Syringa* |
| *S. subbaumii* | Shanxi, Jincheng, Qinshui, Lishan Mountain National Nature Reserve | 111°42′00″ | 35°18′00″ | Yuan 1038 (IFP) | ^ | angiosperm |
| *S. subbaumii* | Shanxi, Jincheng, Qinshui, Lishan Mountain National Nature Reserve | 111°42′00″ | 35°18′00″ | Yuan 2418 (IFP) | ^ | angiosperm |
| *S. subbaumii* | Shanxi, Jincheng, Qinshui, Lishan Mountain National Nature Reserve | 111°42′00″ | 35°18′00″ | Yuan 2444 (IFP) | JX069837 | angiosperm |
| *S. subbaumii* | Shanxi, Jincheng, Qinshui, Lishan Mountain National Nature Reserve | 111°42′00″ | 35°18′00″ | Yuan 2449 (IFP) | & | angiosperm |
| *S. subbaumii* | Shanxi, Luliang, Jiaocheng, Pangquangou, Shanshui | 111°30′00″ | 37°48′00″ | Dai 13360 (BJFC) | MT343580 | *Prunus* |
| *S. vaninii* | Heilongjiang, Yichun, Wuying, Fenglin National Nature Reserve | 129°12′00″ | 48°6′00″ | Dai 3624 (IFP) | JN642590 | *Pinus* |
| *S. vaninii* | Heilongjiang, Yichun, Wuying, Fenglin National Nature Reserve | 129°12′09″ | 48°8′20″ | Dai 3677 (IFP) | ^ | *Populus* |
| *S. vaninii* | Jilin, Yanbian, Antu, Changbai Mountain National Nature Reserve | 128°11′02″ | 42°11′22″ | Cui 9939 (BJFC) | MF772792 |  |
| *S. vaninii* | Jilin, Yanbian, Antu, Changbai Mountain National Nature Reserve | 128°12′00″ | 42°12′00″ | Cui 9965 (BJFC) | ^ | *Populus* |
| *S. vaninii* | Jilin, Yanbian, Antu, Changbai Mountain National Nature Reserve | 128°12′00″ | 42°12′00″ | Cui 9974 (BJFC) | & |  |
| *S. vaninii* | Jilin, Yanbian, Antu, Changbai Mountain National Nature Reserve | 128°12′00″ | 42°12′00″ | Cui 14082 (BJFC) | MF772793 | *Populus* |
| *S. vaninii* | Jilin, Yanbian, Antu, Changbai Mountain National Nature Reserve | 128°12′00″ | 42°12′00″ | Dai 1980 (IFP) | & | *Populus* |
| *S. vaninii* | Jilin, Yanbian, Antu, Changbai Mountain National Nature Reserve | 128°12′00″ | 42°12′00″ | Dai 2102 (BJFC) | ^ | *Populus* |
| *S. vaninii* | Jilin, Yanbian, Antu, Changbai Mountain National Nature Reserve | 128°12′00″ | 42°12′00″ | Dai 7011 (IFP) | JN642591 | *Populus* |
| *S. vaninii* | Jilin, Yanbian, Antu, Changbai Mountain National Nature Reserve | 128°12′00″ | 42°12′00″ | Dai 9061 (BJFC) | & |  |
| *S. vaninii* | Jilin, Yanbian, Antu, Changbai Mountain National Nature Reserve | 128°12′00″ | 42°12′00″ | Dai 11368 (BJFC) | ^ | *Populus* |
| *S. vaninii* | Jilin, Yanbian, Antu, Changbai Mountain National Nature Reserve, Birch Forest Sample Site | 127°6′00″ | 42°18′00″ | Wei 2582 (IFP) | ^ | *Acer* |
| *S. vaninii* | Jilin, Yanbian, Antu, Changbai Mountain National Nature Reserve, Birch Forest Sample Site | 127°6′00″ | 42°18′00″ | Wei 2956 (IFP) | & | angiosperm |
| *S. vaninii* | Jilin, Yanbian, Antu, Changbai Mountain National Nature Reserve, Huangsongpu | 128°12′00″ | 42°12′00″ | Dai 8213 (IFP) | ^ | *Populus* |
| *S. vaninii* | Jilin, Yanbian, Antu, Changbai Mountain National Nature Reserve, Huangsongpu | 128°12′00″ | 42°12′00″ | Dai 8236 (IFP) | MF772791 | *Populus* |
| *S. vaninii* | Jilin, Yanbian, Antu, Changbai Mountain National Nature Reserve, Huangsongpu | 128°12′00″ | 42°12′00″ | Dai 8244 (IFP) | ^ | *Populus* |
| *S. vaninii* | Jilin, Yanbian, Antu, Changbai Mountain National Nature Reserve, Huangsongpu | 128°12′00″ | 42°12′00″ | Dai 8245 (IFP) | & | *Populus* |
| *S. vaninii* | Jilin, Yanbian, Antu, Changbai Mountain National Nature Reserve, Huangsongpu | 128°12′00″ | 42°12′00″ | Wei 3382 (IFP) | JN169788 |  |
| *S. vaninii* | Jilin, Yanbian, Antu, Changbai Mountain National Nature Reserve, Huangsongpu | 128°12′00″ | 42°12′00″ | Yuan 5604 (IFP) | KY328307 | *Quercus* |
| *S. vaninii* | Jilin, Yanbian, Antu, Changbai Mountain National Nature Reserve, Opposite Guangming Forest Farm | 128°12′00″ | 42°12′00″ | Dai 7065 (IFP) | ^ | *Populus* |
| *S. vaninii* | Liaoning, Benxi, Guanmen Mountain National Forest Park | 124°12′00″ | 41°6′00″ | Yuan 3935 (IFP) | & | *Quercus* |
| *S. vaninii* | Shaanxi, Hanzhong, Foping, Foping National Nature Reserve | 108°0′00″ | 33°30′00″ | Yuan 2764 (IFP) | KY328308 | *Quercus* |
| *S. vaninii* | Shaanxi, Hanzhong, Foping, Foping National Nature Reserve | 108°0′00″ | 33°30′00″ | Yuan 2858 (IFP) | ^ | *Populus* |
| *S. vaninii* | Shaanxi, Hanzhong, Foping, Foping National Nature Reserve | 108°0′00″ | 33°30′00″ | Yuan 2868 (IFP) | ^ | angiosperm |
| *S. vitexicola* | Taiwan, Pingdong, Mangchou, Yongjing | 120°46′00″ | 22°1′00″ | Wu 2004-2 | MT906616 | *Vitex negundo* |
| *S. vitexicola* | Taiwan, Pingdong, Mangchou, Yongjing | 120°46′00″ | 22°1′00″ | Wu 2004-3 | MT906617 | *Vitex negundo* |
| *S. vitexicola* | Taiwan, Pingdong, Mangchou, Yongjing | 120°46′00″ | 22°1′00″ | Wu 2005-1 | MT906618 | *Vitex negundo* |
| *S. vitexicola* | Taiwan, Pingdong, Mangchou, Yongjing | 120°46′00″ | 22°1′00″ | Wu 2005-2 | MT906619 | *Vitex negundo* |
| *S. vitexicola* | Taiwan, Pingdong, Mangchou, Yongjing | 120°46′00″ | 22°1′00″ | Wu 2006-21 | MT906620 | *Vitex negundo* |
| *S. vitexicola* | Taiwan, Pingdong, Mangchou, Yongjing | 120°46′00″ | 22°1′00″ | Wu 2006-71 | MT906621 | *Vitex negundo* |
| *S. weigelae* | Anhui, Huangshan, Huangshan Mountain | 118°12′00″ | 30°6′00″ | Dai 6179 (BJFC) | ^ |  |
| *S. weigelae* | Chongqing, Nanchuan, Jinfo Mountain National Nature Reserve | 107°6′00″ | 29°0′00″ | Dai 15768 (BJFC) | & | *Weigela* |
| *S. weigelae* | Chongqing, Nanchuan, Jinfo Mountain National Nature Reserve | 107°6′00″ | 29°0′00″ | Dai 15770 (BJFC) | MF772795 | *Weigela* |
| *S. weigelae* | Chongqing, Nanchuan, Jinfo Mountain National Nature Reserve | 107°6′00″ | 29°0′00″ | Dai 15773 (BJFC) | & | *Weigela* |
| *S. weigelae* | Guizhou, Qiandongnan, Leishan, Leigong Mountain National Nature Reserve | 108°12′00″ | 26°24′00″ | Dai 16531 (BJFC) | & | *Weigela* |
| *S. weigelae* | Guizhou, Qiandongnan, Leishan, Leigong Mountain National Nature Reserve | 108°12′00″ | 26°24′00″ | Dai 16532 (BJFC) | & | *Weigela* |
| *S. weigelae* | Guizhou, Tongren, Jiangkou, Fanjing Mountain National Nature Reserve | 108°48′00″ | 27°48′00″ | Dai 14993 (BJFC) | & | *Weigela* |
| *S. weigelae* | Guizhou, Tongren, Jiangkou, Fanjing Mountain National Nature Reserve | 108°48′00″ | 27°48′00″ | Yuan 5526 (BJFC) | JN169786 | angiosperm |
| *S. weigelae* | Guizhou, Zunyi, Suiyang, Kuankuoshu National Nature Reserve | 107°12′00″ | 28°0′00″ | Dai 3212 (IFP) | ^ | angiosperm |
| *S. weigelae* | Hebei, Chengde, Xinglong, Wuling Mountain National Nature Reserve | 117°24′00″ | 40°36′00″ | Cui 7176 (BJFC) | JQ860320 | *Syringa* |
| *S. weigelae* | Hebei, Chengde, Xinglong, Wuling Mountain National Nature Reserve | 117°24′00″ | 40°36′00″ | Cui 7192 (BJFC) | ^ |  |
| *S. weigelae* | Hubei, Yichang, Wufeng, Houhe National Nature Reserve | 110°36′00″ | 30°6′00″ | Wei 2267 (IFP) | JX069834 | angiosperm |
| *S. weigelae* | Hubei, Yichang, Wufeng, Houhe National Nature Reserve | 110°36′00″ | 30°6′00″ | Wei 2345 (IFP) | ^ | *Quercus* |
| *S. weigelae* | Hubei, Yichang, Xingshan, Longmenhe National Forest Park | 110°30′00″ | 31°18′00″ | Wei 2073 (BJFC) | ^ | *Coriaria* |
| *S. weigelae* | Hubei, Yichang, Xingshan, Longmenhe National Forest Park | 110°30′00″ | 31°18′00″ | Wei 2120 (IFP) | JQ860314 | *Coriaria* |
| *S. weigelae* | Hunan, Changde, Shimen, Huping Mountain Nature Reserve | 110°48′00″ | 25°54′00″ | Dai 12139 (BJFC) | ^ |  |
| *S. weigelae* | Hunan, Changsha, Liuyang, Dawei Mountain National Forest Park | 114°6′00″ | 28°6′00″ | Dai 3276 (IFP) | ^ | angiosperm |
| *S. weigelae* | Hunan, Yizhou, Yizhang, Mangshan Mountain National Nature Reserve | 112°54′00″ | 24°54′00″ | Li 1809 (IFP) | ^ | angiosperm |
| *S. weigelae* | Hunan, Zhangjiajie National Forest Park | 110°24′00″ | 29°18′00″ | Dai 11694 (BJFC) | JQ860315 |  |
| *S. weigelae* | Jiangxi, Jiujiang, Lushan Mountain | 116°0′00″ | 29°36′00″ | Cui 6001 (BJFC) | & | *Lonicera* |
| *S. weigelae* | Jiangxi, Jiujiang, Lushan Mountain | 116°0′00″ | 29°36′00″ | Cui 6010 (BJFC) | JQ860318 | *Lonicera* |
| *S. weigelae* | Jiangxi, Jiujiang, Lushan Mountain | 116°0′00″ | 29°36′00″ | Cui 6012 (BJFC) | JQ860319 | *Lonicera* |
| *S. weigelae* | Jiangxi, Jiujiang, Lushan Mountain, Trinity Spring | 116°3′03″ | 29°33′43″ | LWZ 20150802-3 (IFP) | MT348590 | angiosperm |
| *S. weigelae* | Jiangxi, Jiujiang, Lushan Mountain, Trinity Spring | 116°3′03″ | 29°33′43″ | LWZ 20150802-5 (IFP) | MT348591 | angiosperm |
| *S. weigelae* | Jiangxi, Jiujiang, Lushan Mountain, Trinity Spring | 116°3′03″ | 29°33′43″ | LWZ 20210623-1a (HMAS) | ^ | angiosperm |
| *S. weigelae* | Jiangxi, Jiujiang, Lushan Mountain, Trinity Spring | 116°3′03″ | 29°33′43″ | LWZ 20210623-2a (HMAS) | ^ | angiosperm |
| *S. weigelae* | Jiangxi, Jiujiang, Lushan Mountain, Trinity Spring | 116°3′03″ | 29°33′43″ | LWZ 20210623-20a (HMAS) | ^ | angiosperm |
| *S. weigelae* | Jiangxi, Jiujiang, Lushan Mountain, Trinity Spring | 116°3′03″ | 29°33′43″ | LWZ 20210623-21a (HMAS) | ^ | angiosperm |
| *S. weigelae* | Neimenggu, Chifeng, Ningcheng, Heilihe National Nature Reserve, Daxugou | 118°24′00″ | 41°36′00″ | Dai 16064 (BJFC) | & | *Weigela* |
| *S. weigelae* | Neimenggu, Chifeng, Ningcheng, Heilihe National Nature Reserve, Daxugou | 118°24′00″ | 41°36′00″ | Dai 16069 (BJFC) | & | *Weigela* |
| *S. weigelae* | Neimenggu, Chifeng, Ningcheng, Heilihe National Nature Reserve, Dabagou | 118°24′00″ | 41°36′00″ | Dai 16072 (BJFC) | MT348589 | *Weigela* |
| *S. weigelae* | Neimenggu, Chifeng, Ningcheng, Heilihe National Nature Reserve, Dabagou | 118°24′00″ | 41°36′00″ | Dai 16074 (BJFC) | & | *Weigela* |
| *S. weigelae* | Neimenggu, Chifeng, Ningcheng, Heilihe National Nature Reserve, Dabagou | 118°24′00″ | 41°36′00″ | Dai 16076 (BJFC) | ^ | *Weigela* |
| *S. weigelae* | Neimenggu, Chifeng, Ningcheng, Heilihe National Nature Reserve, Dabagou | 118°24′00″ | 41°36′00″ | Dai 16077 (BJFC) | MF772794 | *Weigela* |
| *S. weigelae* | Zhejiang, Hangzhou, Lin'an, Tianmu Mountain National Nature Reserve | 119°24′00″ | 30°18′00″ | Cui 2693 (BJFC) | ^ |  |
| *S. weigelae* | Zhejiang, Hangzhou, Lin'an, Tianmu Mountain National Nature Reserve | 119°24′00″ | 30°18′00″ | Cui 2764 (BJFC) | ^ |  |
| *S. weigelae* | Zhejiang, Hangzhou, Lin'an, Tianmu Mountain National Nature Reserve | 119°24′00″ | 30°18′00″ | Dai 6312 (BJFC) | ^ | *Sambucus* |
| *S. weigelae* | Zhejiang, Hangzhou, Lin'an, Tianmu Mountain National Nature Reserve | 119°24′00″ | 30°18′00″ | Dai 6352 (BJFC) | JQ860317 |  |
| *S. zonatus* | Hainan, Ledong, Jianfengling National Nature Reserve | 108°48′00″ | 18°42′00″ | Cui 6631 (BJFC) | JQ860305 | angiosperm |
| *S. zonatus* | Hainan, Ledong, Jianfengling National Nature Reserve | 108°48′00″ | 18°42′00″ | Dai 10841 (BJFC) | JQ860306 | angiosperm |
| *S. zonatus* | Yunnan, Kuming, Heilongtan Park | 102°48′00″ | 25°6′00″ | Cui 8327 (BJFC) | JX069837 | angiosperm |
| *S.* sp. | Gansu, Pingliang, Kongtong Mountain Scenic Spot | 106°30′00″ | 35°36′00″ | Yuan 6538 (IFP) | & | *Syringa* |
| *S.* sp. | Gansu, Pingliang, Kongtong Mountain Scenic Spot | 106°30′00″ | 35°36′00″ | Yuan 6564 (IFP) | & | *Syringa* |
| *S.* sp. | Guangxi, Laibin, Jinxiu, Dayao Mountain | 110°12′00″ | 24°6′00″ | BJFC 001641 | ^ |  |
| *S.* sp. | Yunnan, Pu'er, Jingdong, Ailao Mountain National Nature Reserve | 101°12′00″ | 24°30′00″ | Yuan 3312 (BJFC) | ^ |  |

^#^ The accession number of sequence from ITS gene if available in GenBank is directly provided; & means that sequence from other genes but ITS gene is available in GenBank, while ^ means that sequence from any gene is unavailable in GenBank.
