# Supplementary material for: Modeling current geographic distribution and future range shifts of Sanghuangporus under multiple climate change scenarios in China
Source: Front Microbiol. 2022 Dec 1;13:1064451. doi: 10.3389/fmicb.2022.1064451 (PMC9751338; doi:10.3389/fmicb.2022.1064451)
Supplement: Supplementary file 2 [file Table_3.DOCX]

Table S2 Area and variation of the predicted potential distribution for the entire genus *Sanghuangporus* at different suitability level

| Period | Scenario | Area (10^4^ km^2^) / Variation comparing with the current area (%) | | | |
| --- | --- | --- | --- | --- | --- |
|  |  | Unsuitability | Low suitability | Moderate suitability | High suitability |
| current | – | 671.543/– | 182.341/– | 91.283/– | 20.238/– |
| current~ | – | 623.073/-7.218 | 184.994/1.455 | 105.637/15.725 | 51.701/155.468 |
| 2030s | SSP1-2.6 | 644.813/-3.980 | 170.649/-6.413 | 112.887/23.667 | 37.056/83.104 |
|  | SSP2-4.5 | 631.020/-6.034 | 172.603/-5.341 | 127.625/39.813 | 34.158/68.781 |
|  | SSP3-7.0 | 611.943/-8.875 | 164.605/-9.727 | 147.201/61.258 | 41.657/105.837 |
|  | SSP5-8.5 | 647.284/-3.612 | 175.137/-3.951 | 109.151/19.575 | 33.833/67.179 |
| 2050s | SSP1-2.6 | 614.817/-8.447 | 179.040/-1.810 | 129.723/42.111 | 41.825/106.666 |
|  | SSP2-4.5 | 616.922/-8.134 | 169.007/-7.313 | 135.306/48.227 | 44.170/118.255 |
|  | SSP3-7.0 | 642.732/-4.290 | 172.716/-5.279 | 111.400/22.038 | 38.558/90.525 |
|  | SSP5-8.5 | 657.832/-2.042 | 160.959/-11.727 | 105.551/15.630 | 41.064/102.909 |
| 2070s | SSP1-2.6 | 637.439/-5.078 | 170.884/-6.284 | 113.422/24.253 | 43.660/115.734 |
|  | SSP2-4.5 | 633.336/-5.689 | 169.520/-7.032 | 119.767/31.204 | 42.783/111.400 |
|  | SSP3-7.0 | 649.778/-3.241 | 172.610/-5.337 | 106.206/16.348 | 36.812/81.896 |
|  | SSP5-8.5 | 628.932/-6.345 | 171.641/-5.869 | 123.296/35.070 | 41.537/105.243 |
| 2090s | SSP1-2.6 | 622.250/-7.340 | 180.912/-0.784 | 122.419/34.109 | 39.824/ 96.779 |
|  | SSP2-4.5 | 606.227/-9.726 | 184.457/1.160 | 134.795/47.667 | 39.926/97.286 |
|  | SSP3-7.0 | 646.216/-3.772 | 175.637/-3.677 | 107.733/18.021 | 35.820/76.996 |
|  | SSP5-8.5 | 595.079/-11.386 | 190.695/4.581 | 137.223/50.327 | 42.408/109.549 |

The tilde (~) means that Host plant is excluded from the environmental variables for modeling.
