# Supplementary material for: Modeling current geographic distribution and future range shifts of Sanghuangporus under multiple climate change scenarios in China
Source: Front Microbiol. 2022 Dec 1;13:1064451. doi: 10.3389/fmicb.2022.1064451 (PMC9751338; doi:10.3389/fmicb.2022.1064451)
Supplement: Supplementary file 3 [file Table_4.DOCX]

Table S3 The coordinate and migration distance of mass center of the predicted potential distribution for the entire genus *Sanghuangporus*

| Period | Scenario | Longitude | Latitude | Migration distance comparing with the current coordinate (km) |
| --- | --- | --- | --- | --- |
| current | – | 116°24′24″ | 36°34′34″ |  |
| current~ | – | 113°58′52″ | 35°17′26″ | 261.312 |
| 2030s | SSP1-2.6 | 115°21′48″ | 35°24′12″ | 160.858 |
|  | SSP2-4.5 | 117°54′56″ | 36°47′07″ | 136.696 |
|  | SSP3-7.0 | 115°42′15″ | 36°1′38″ | 87.783 |
|  | SSP5-8.5 | 118°18′44″ | 37°18′31″ | 188.108 |
| 2050s | SSP1-2.6 | 117°23′18″ | 36°54′21″ | 94.941 |
|  | SSP2-4.5 | 114°0′32″ | 35°55′24″ | 227.195 |
|  | SSP3-7.0 | 117°47′48″ | 36°44′26″ | 125.469 |
|  | SSP5-8.5 | 117°49′27″ | 37°22′41″ | 154.464 |
| 2070s | SSP1-2.6 | 114°47′49″ | 36°46′02″ | 145.289 |
|  | SSP2-4.5 | 118°27′42″ | 37°20′11″ | 201.419 |
|  | SSP3-7.0 | 116°6′37″ | 36°29′54″ | 27.892 |
|  | SSP5-8.5 | 117°33′33″ | 37°10′46″ | 122.638 |
| 2090s | SSP1-2.6 | 117°6′37″ | 36°52′02″ | 70.649 |
|  | SSP2-4.5 | 117°39′49″ | 38°15′13″ | 217.287 |
|  | SSP3-7.0 | 119°7′19″ | 38°40′18″ | 334.202 |
|  | SSP5-8.5 | 115°19′50″ | 36°9′23″ | 107.196 |

The tilde (~) means that Host plant is excluded from the environmental variables for modeling.
