# Supplementary material for: Modeling current geographic distribution and future range shifts of Sanghuangporus under multiple climate change scenarios in China
Source: Front Microbiol. 2022 Dec 1;13:1064451. doi: 10.3389/fmicb.2022.1064451 (PMC9751338; doi:10.3389/fmicb.2022.1064451)
Supplement: Supplementary file 4 [file Data_Sheet_1.docx]

***Supplementary Material***

1. **Supplementary Figures and Tables**

**2.1 Supplementary Figures**


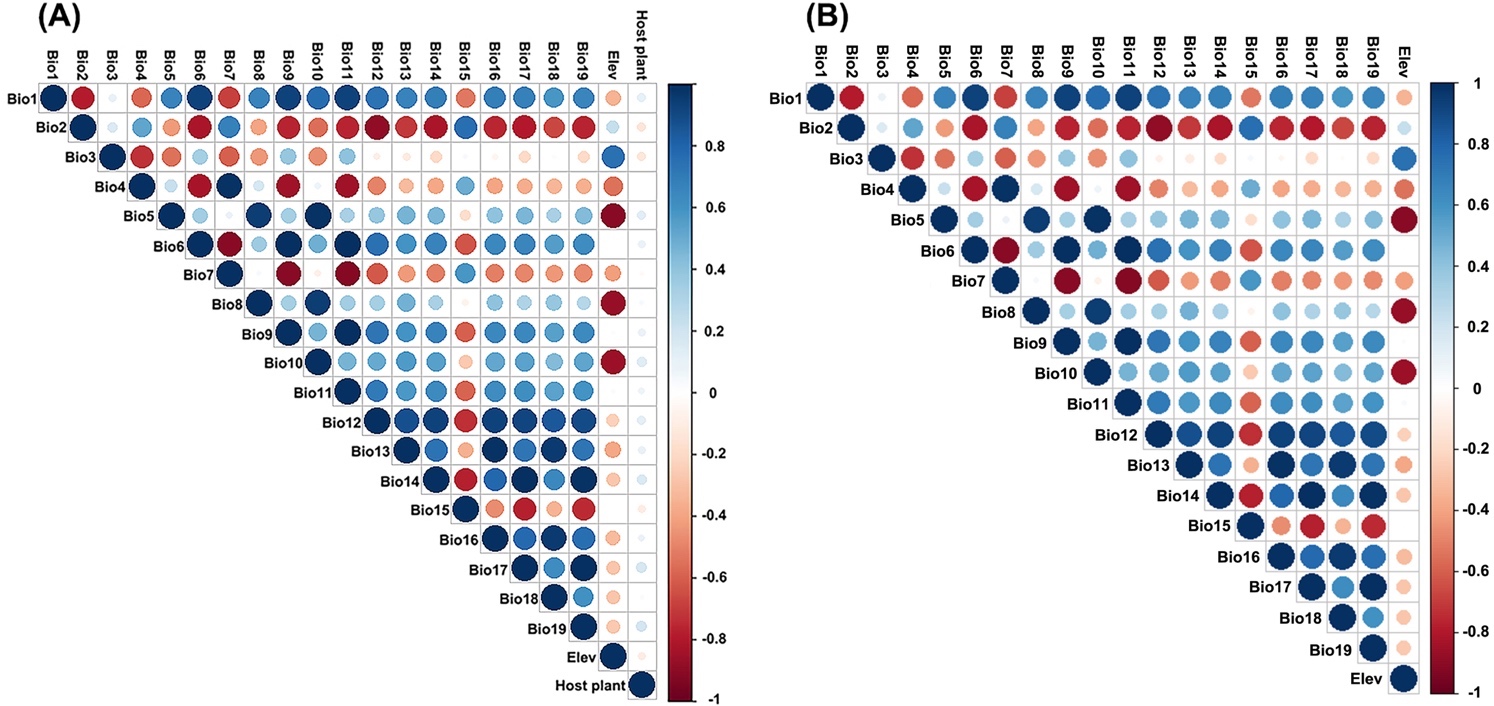


**Supplementary Figure 1.** Correlation of environmental variables for modeling the potential distribution of *Sanghuangporus* with the inclusion of Host plant in the environmental variables (A) or not (B).


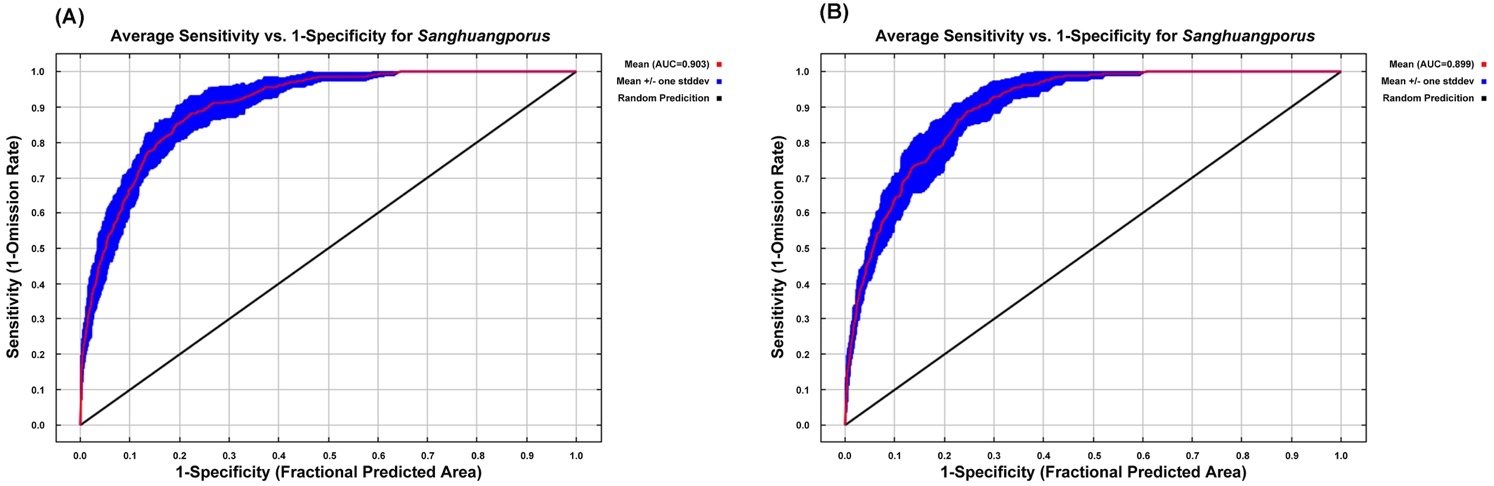


**Supplementary Figure 2.** A receiver operating characteristic curve and the value of Area Under Receiver Operator Characteristic Curve under the current period (10 replicated runs) with the inclusion of Host plant in the environmental variables (A) or not (B).


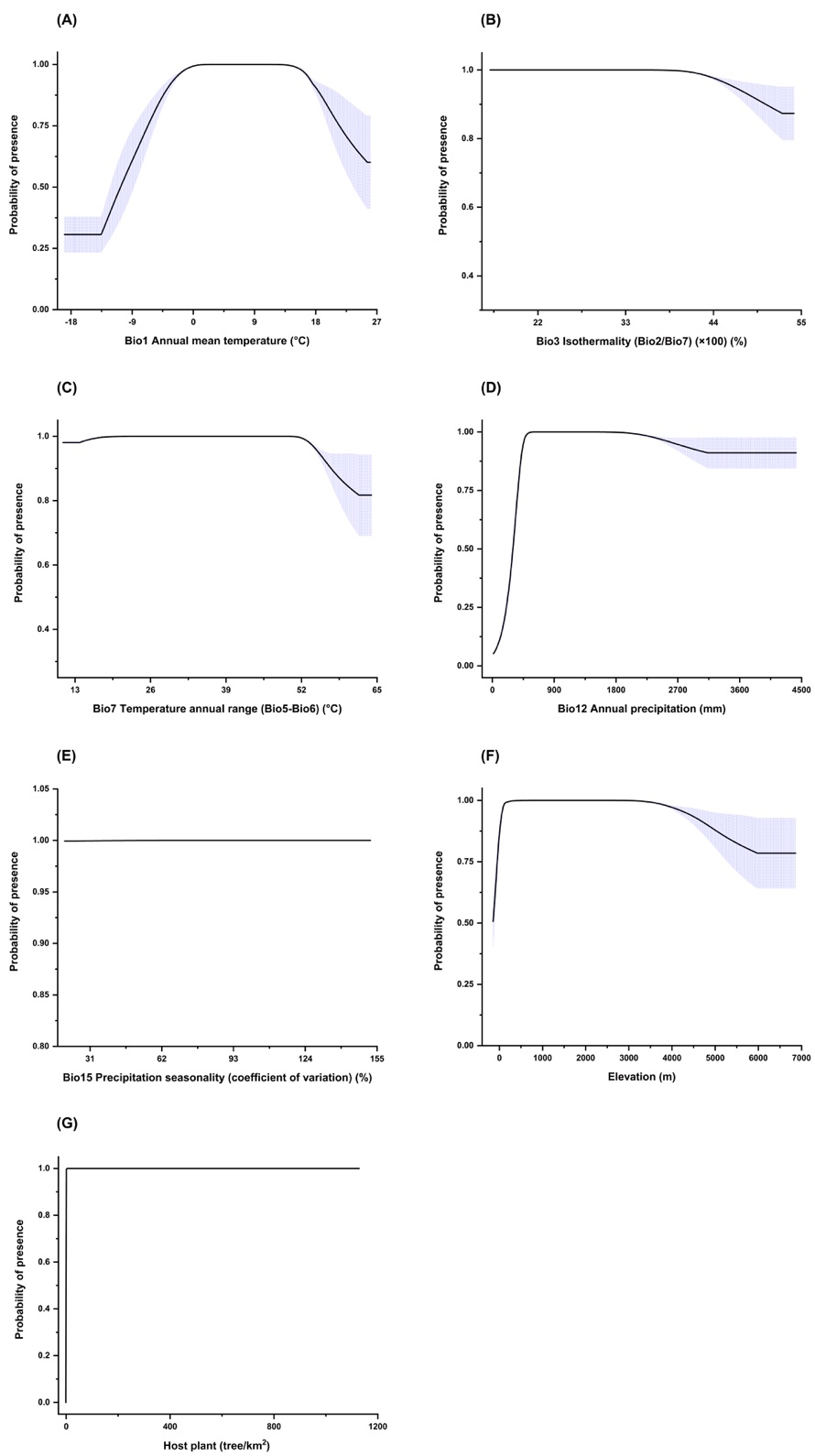
**Supplementary Figure 3.** Response curves of the critical environmental variables to the distribution model of Sanghuangporus. (A) Bio1 Annual mean temperature, (B) Bio3 Isothermality (Bio2/Bio7) (×100), (C) Bio7 Temperature annual range (Bio5-Bio6), (D) Bio12 Annual precipitation, (E) Bio15 Precipitation seasonality (coefficient of variation), (F) Elevation and (G) Host plant.

**
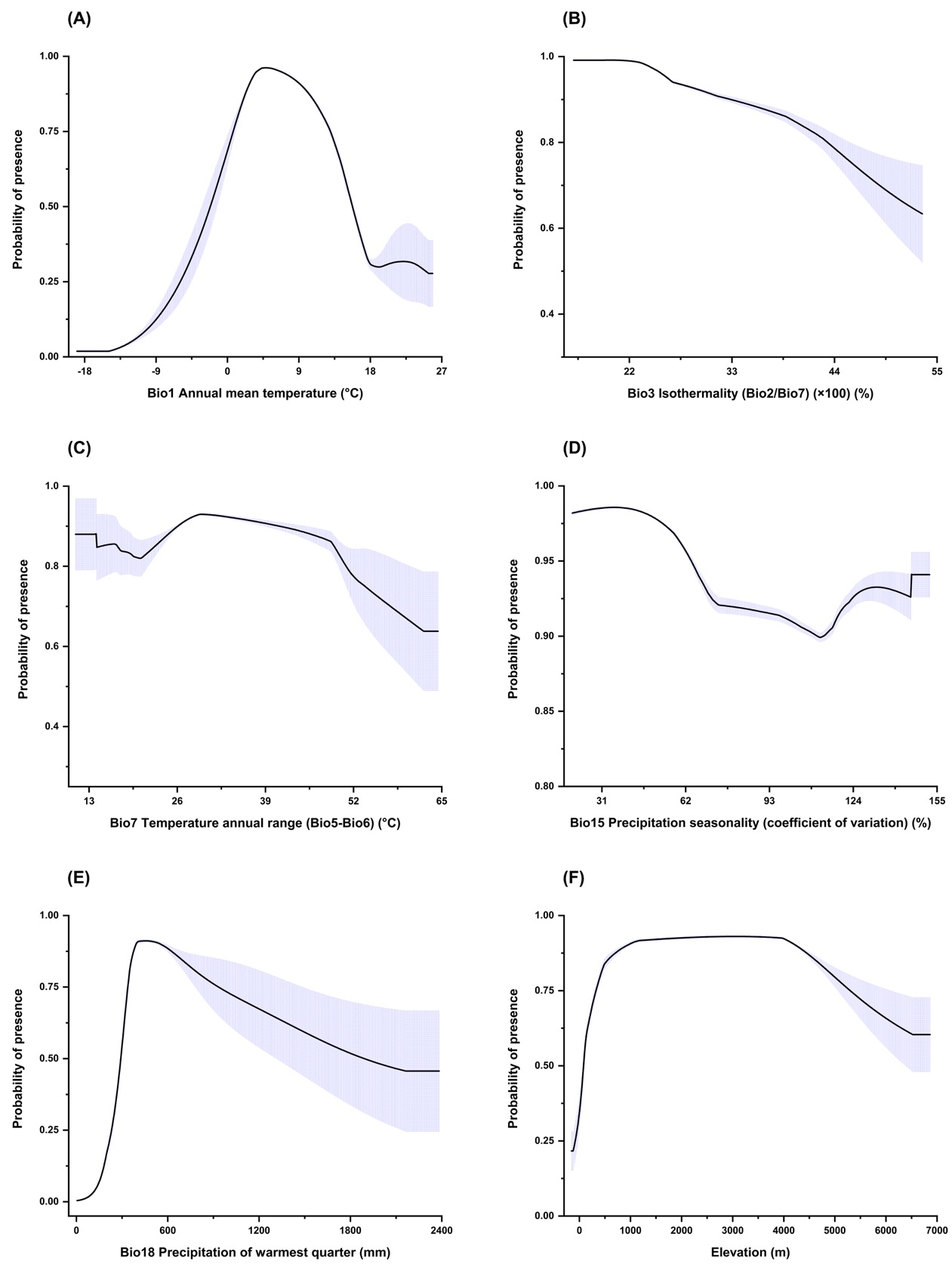
Supplementary Figure 4.** Response curves of the critical environmental variables to the distribution model of Sanghuangporus with the exclusion of Host plant from the environmental variables. (A) Bio1 Annual mean temperature, (B) Bio3 Isothermality (Bio2/Bio7) (×100), (C) Bio7 Temperature annual range (Bio5-Bio6), (D) Bio15 Precipitation seasonality (coefficient of variation), (E) Bio18 Precipitation of warmest quarter and (F) Elevation.


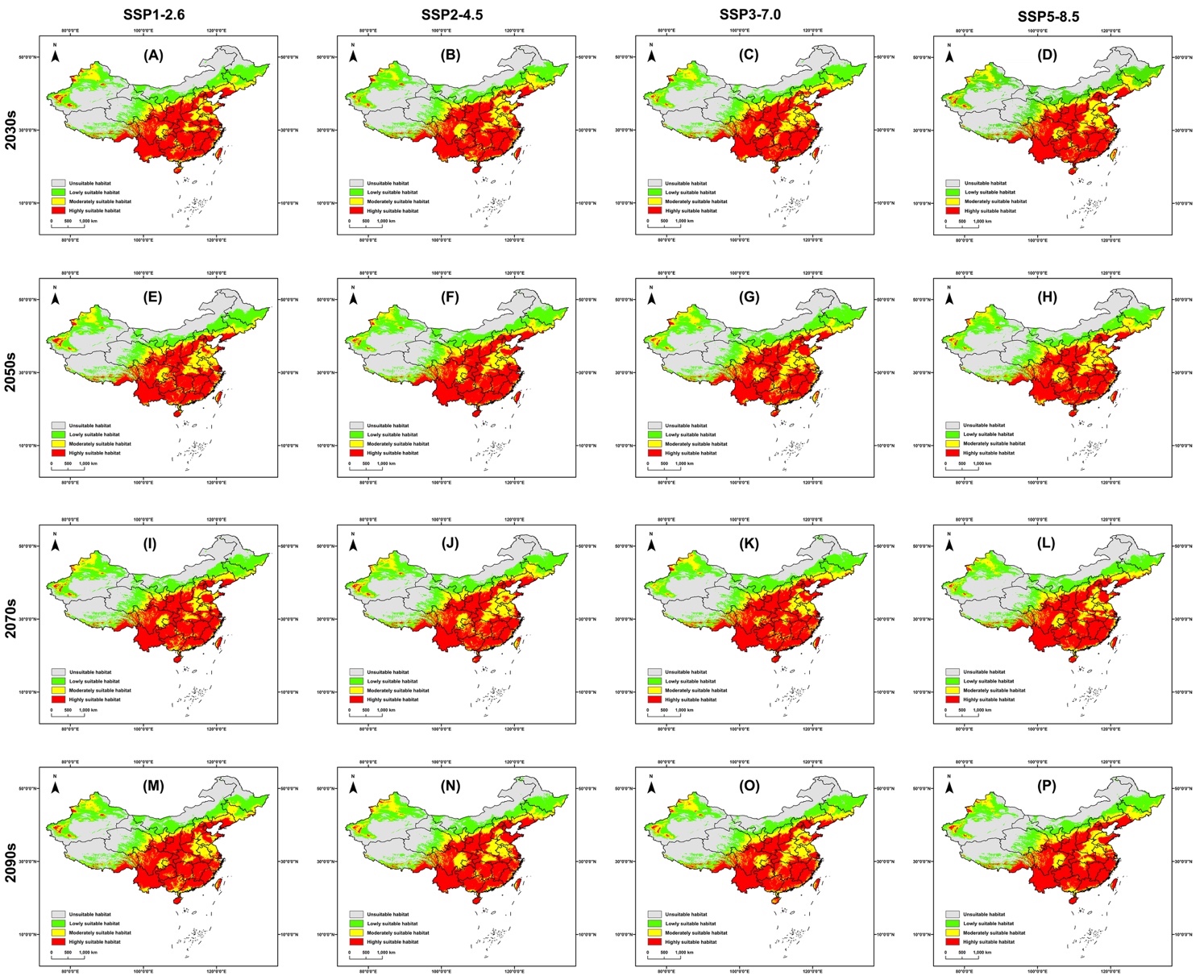
**Supplementary Figure 5.** Distribution models of host plants of *Sanghuangporus* under future scenarios in China. (A) Under SSP1-2.6 scenario in the 2030s, (B) Under SSP2-4.5 scenario in the 2030s, (C) Under SSP3-7.0 scenario in the 2030s, (D) Under SSP5-8.5 scenario in the 2030s, (E) Under SSP1-2.6 scenario in the 2050s, (F) Under SSP2-4.5 scenario in the 2050s, (G) Under SSP3-7.0 scenario in the 2050s, (H) Under SSP5-8.5 scenario in the 2050s, (I) Under SSP1-2.6 scenario o in the 2070s, (J) Under SSP2-4.5 scenario in the 2070s, (K) Under SSP3-7.0 scenario in the 2070s, (L) Under SSP5-8.5 scenario in the 2070s, (M) Under SSP1-2.6 scenario in the 2090s, (N) Under SSP2-4.5 scenario in the 2090s, (O) Under SSP3-7.0 scenario in the 2090s, (P) Under SSP5-8.5 scenario in the 2090s.
