# Supplementary figures and images for: Modeling current geographic distribution and future range shifts of Sanghuangporus under multiple climate change scenarios in China
Source: Front Microbiol. 2022 Dec 1;13:1064451. doi: 10.3389/fmicb.2022.1064451 (PMC9751338; doi:10.3389/fmicb.2022.1064451)

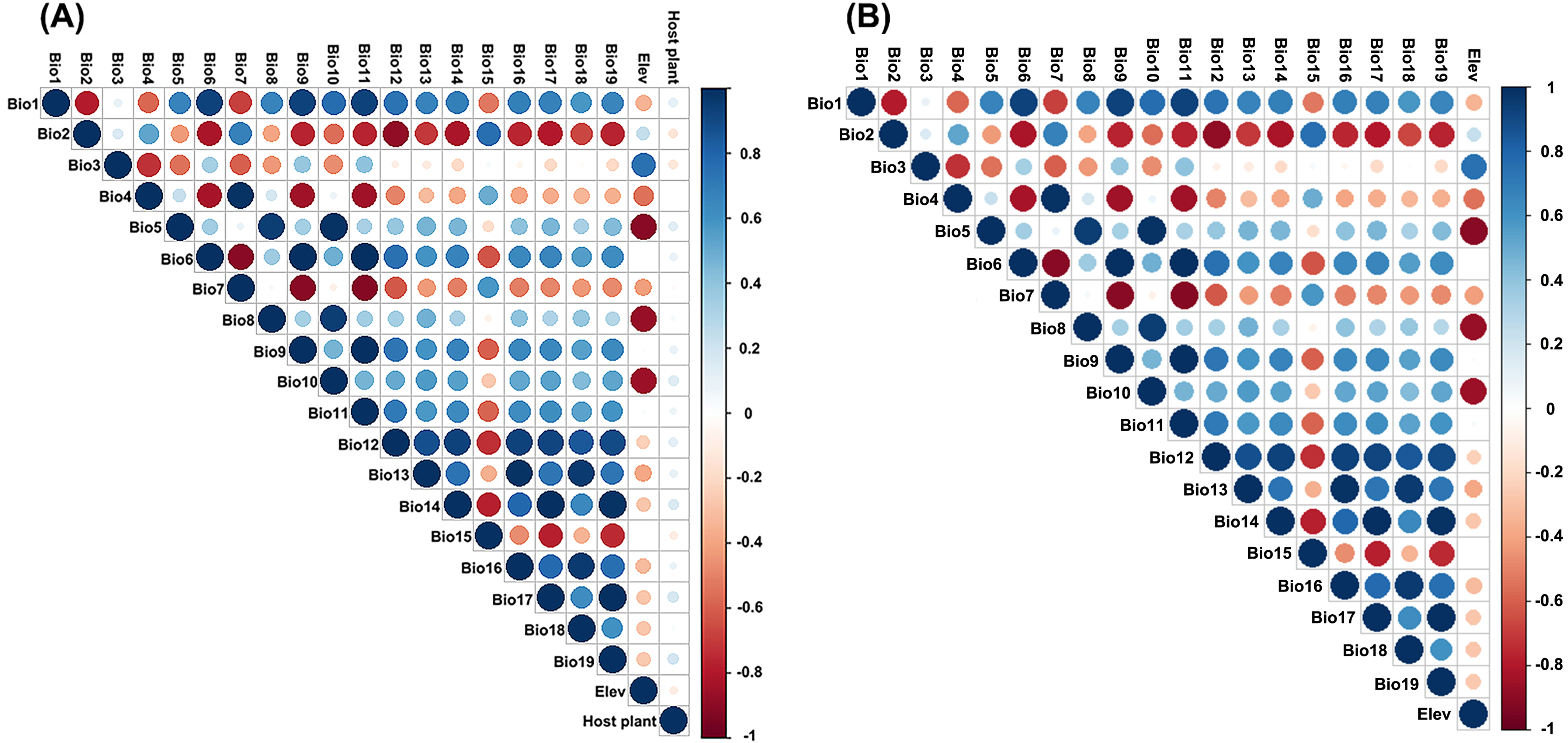

Supplement: Supplementary file 5 [file Image_1.JPEG]

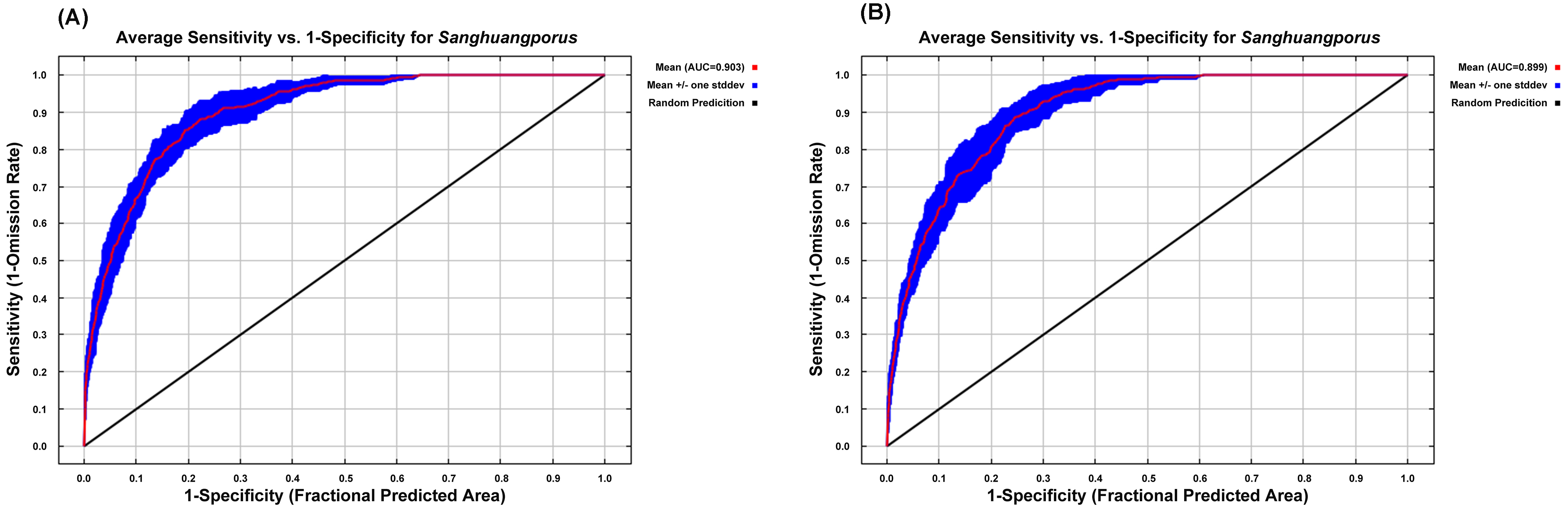

Supplement: Supplementary file 6 [file Image_2.JPEG]

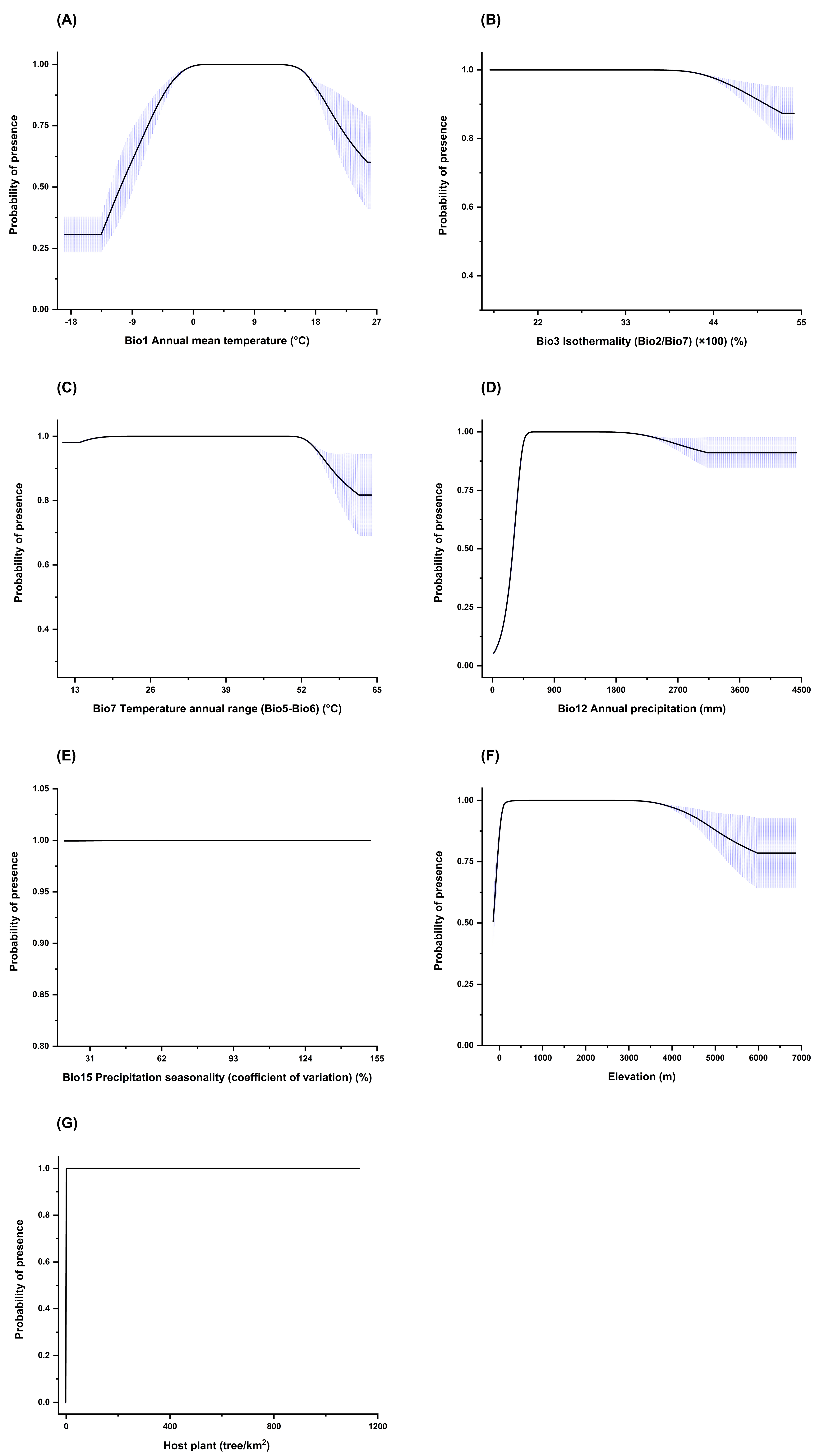

Supplement: Supplementary file 7 [file Image_3.JPEG]

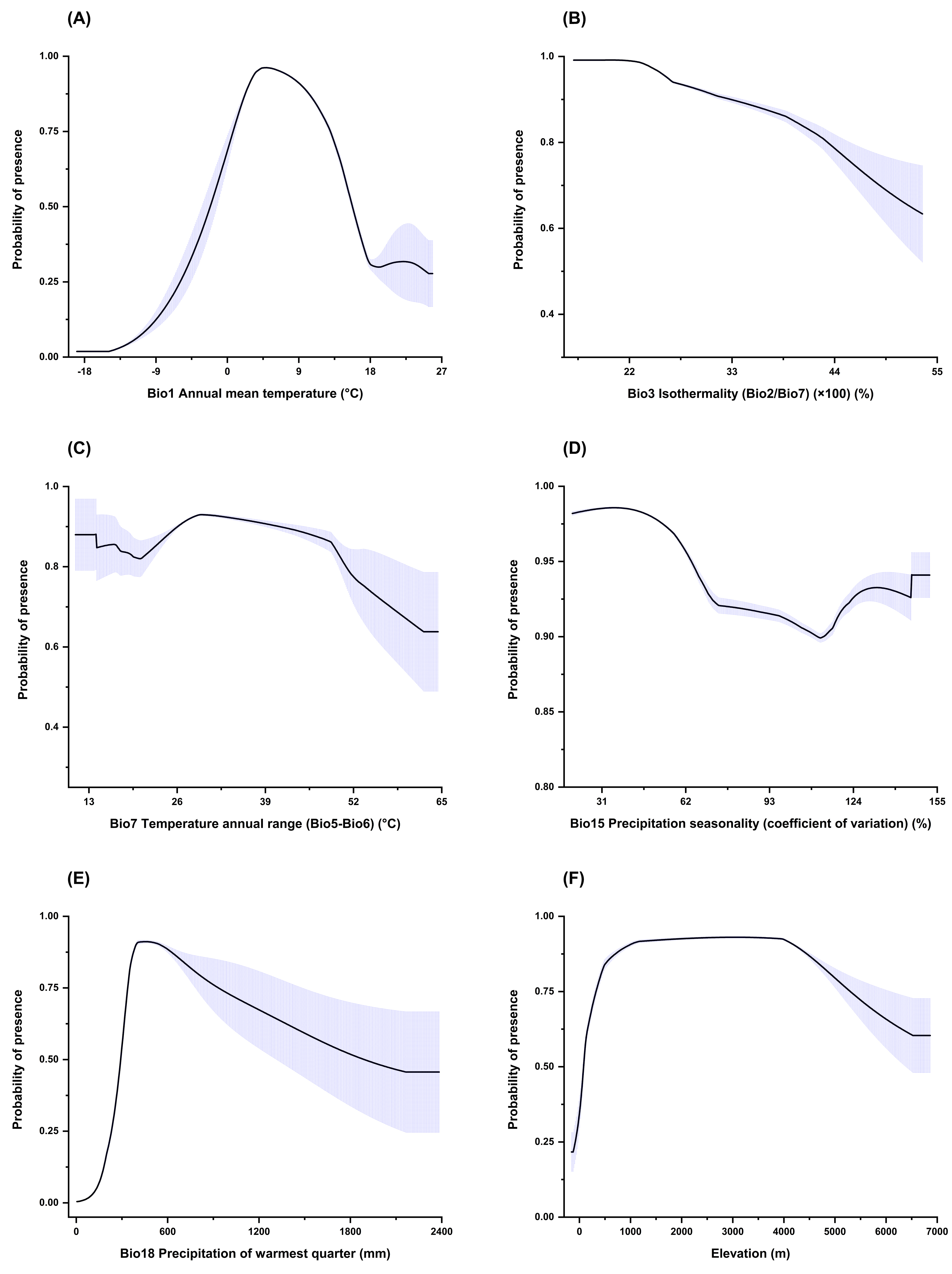

Supplement: Supplementary file 8 [file Image_4.JPEG]

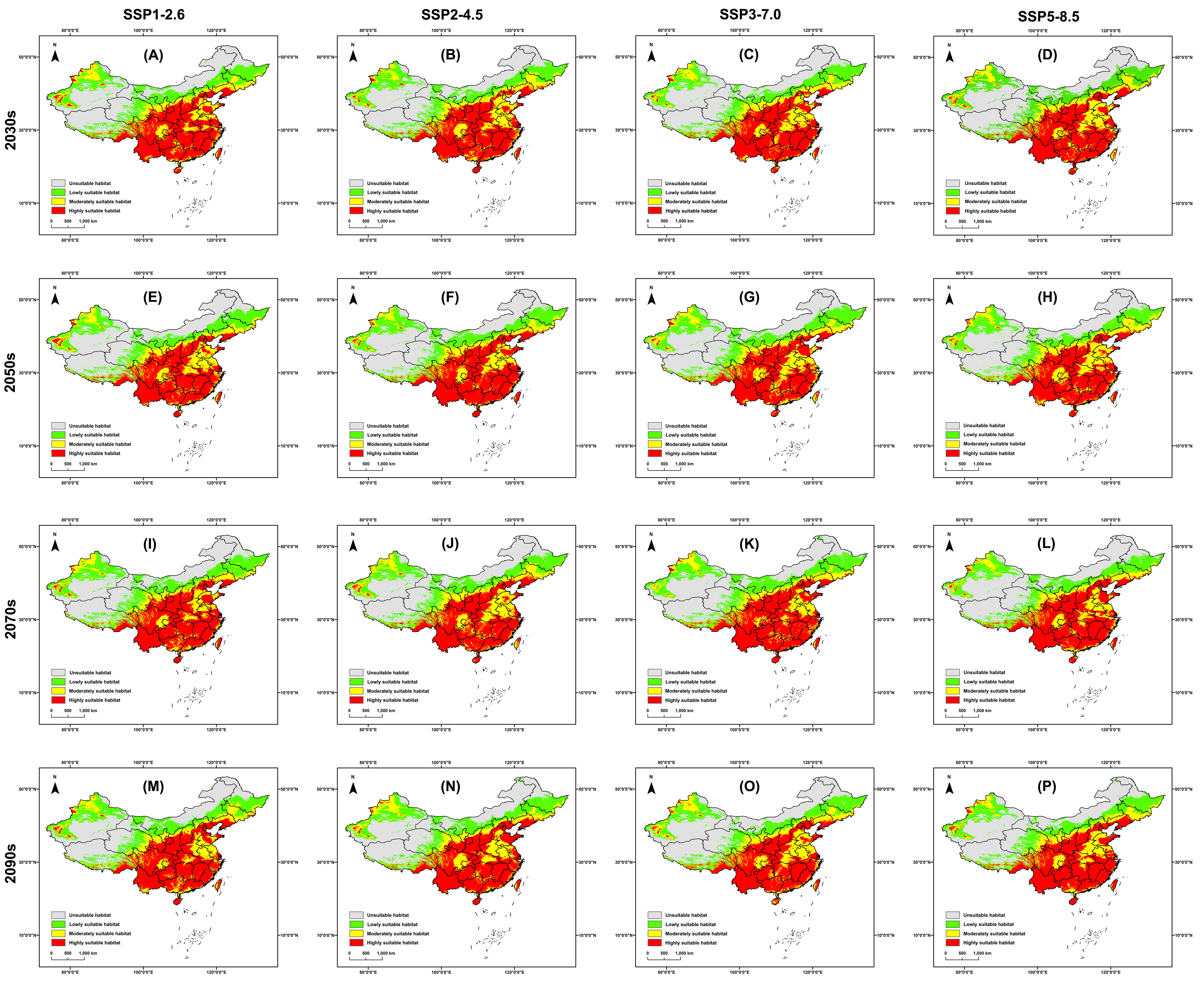

Supplement: Supplementary file 9 [file Image_5.JPEG]
